# A Glucose‐Responsive Hydrogel Inhibits Primary and Secondary BRB Injury for Retinal Microenvironment Remodeling in Diabetic Retinopathy

**DOI:** 10.1002/advs.202402368

**Published:** 2024-06-21

**Authors:** Yue Zhou, Chan Zhao, Zhiyuan Shi, Zbynek Heger, HuaQing Jing, Zhengming Shi, Yunsheng Dou, Siyu Wang, Zitong Qiu, Nan Li

**Affiliations:** ^1^ Tianjin Key Laboratory of Drug Delivery & High‐Efficiency School of Pharmaceutical Science and Technology Tianjin University Tianjin 300072 P. R. China; ^2^ Department of Pharmacy Tianjin Union Medical Center Nankai University Tianjin 300122 P. R. China; ^3^ Department of Ophthalmology Peking Union Medical College Hospital Chinese Academy of Medical Sciences Beijing 100730 P. R. China; ^4^ Key Laboratory of Ocular Fundus Diseases Chinese Academy of Medical Sciences & Peking Union Medical College Beijing 100730 P. R. China; ^5^ Department of Chemistry and Biochemistry Mendel University in Brno Brno CZ‐61300 Czech Republic

**Keywords:** diabetic retinopathy, primary BRB injury, retinal microenvironment reshaping, secondary BRB injury, siMyD88

## Abstract

Current diabetic retinopathy (DR) treatment involves blood glucose regulation combined with laser photocoagulation or intravitreal injection of vascular endothelial growth factor (VEGF) antibodies. However, due to the complex pathogenesis and cross‐interference of multiple biochemical pathways, these interventions cannot block disease progression. Recognizing the critical role of the retinal microenvironment (RME) in DR, it is hypothesized that reshaping the RME by simultaneously inhibiting primary and secondary blood–retinal barrier (BRB) injury can attenuate DR. For this, a glucose‐responsive hydrogel named Cu‐PEI/siMyD88@GEMA‐Con A (CSGC) is developed that effectively delivers Cu‐PEI/siMyD88 nanoparticles (NPs) to the retinal pigment epithelium (RPE). The Cu‐PEI NPs act as antioxidant enzymes, scavenging ROS and inhibiting RPE pyroptosis, ultimately blocking primary BRB injury by reducing microglial activation and Th1 differentiation. Simultaneously, MyD88 expression silence in combination with the Cu‐PEI NPs decreases IL‐18 production, synergistically reduces VEGF levels, and enhances tight junction proteins expression, thus blocking secondary BRB injury. In summary, via remodeling the RME, the CSGC hydrogel has the potential to disrupt the detrimental cycle of cross‐interference between primary and secondary BRB injury, providing a promising therapeutic strategy for DR.

## Graphical abstract

A glucose‐responsive hydrogel, Cu‐PEI/siMyD88@GEMA‐Con A (CSGC), is developed to deliver Cu‐PEI/siMyD88 NPs to the retinal pigment epithelium (RPE) (Figure a). Cu‐PEI acts as an antioxidant, reducing ROS and inhibiting RPE cells' pyroptosis, thus blocking primary blood‐retinal barrier (BRB) injury. Simultaneously, siMyD88 inhibits MyD88 expression, reducing IL‐18 and vascular endothelial growth factor (VEGF) expression, and enhancing tight junction protein expression, thus blocking secondary BRB injury (Figure b). In summary, the CSGC hydrogel efficiently remodels retinal microenvironment (RME), representing a promising diabetic retinopathy (DR) therapy.

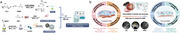

## 1 Introduction

Diabetic retinopathy (DR), one of the major debilitating complications of diabetes, is a significant contributor to the prevalence of blindness in adults worldwide.^[^ ^1^ ^]^ Impairment of the blood‒retinal barrier (BRB), which leads to excessive retinal vascular leakage of plasma, macromolecules, and electrolytes, is the hallmark of the early stages of DR.^[^ ^2^ ^]^ Without effective management, this condition may progress to macular edema and/or advanced microvasculopathy, subsequently reaching the late stages of DR.^[^ ^3^ ^]^ This gradual process impairs retinal neurons and glia, ultimately leading to the distortion and loss of central vision with high‐definition clarity.^[^ ^4^ ^]^ Recent advancements in understanding have revealed that the retinal microenvironment (RME) is a complex interplay of retinal cells, immune cells, the extracellular matrix, soluble molecules, and the retinal vasculature.^[^ ^5^ ^]^ Under normal conditions, these components work together and play integral roles in maintaining retinal homeostasis. However, the dynamic changes within the various components of the RME along with the disruption of retinal homeostasis, underlie the onset of numerous retinal conditions, including DR.^[^ ^6^ ^]^ Therefore, it is important to note the potential for regulating the RME using RME‐specific drugs to preserve or restore retinal homeostasis, thereby preventing or treating DR, representing a significant advancement.

The contribution of the RME to promoting DR can be summarized in two main aspects. First, the abnormal activation of innate immune cells, along with the differentiation of adaptive immune cells triggered by primary BRB injury, sustains the disruption of neurovascular units. Oxidative stress in the retinal pigment epithelium (RPE) and pericytes induces subtle damage to the BRB structure, which is regarded as a primary BRB injury, enhancing the activation of retinal glial cells, including Müller cells and microglia.^[^ ^7^ ^]^ As important innate immune cells, activated microglia drive CD4^+^ T cell differentiation into pro‐inflammatory T helper 1 (Th1) and Th17 cells,^[^ ^8^ ^]^ inducing edema and damage to the neurovascular unit through a cascade reaction. Second, the dysregulation of pro‐inflammatory cytokines and aberrant expression of connexin between epithelial/endothelial cells and the extracellular matrix exacerbate secondary BRB injury.^[^ ^9^ ^]^ Following primary BRB injury, overexpressed pro‐inflammatory factors activate mitogen‐activated protein kinase (MAPK) and nuclear transcription factor (NF‐κB) signaling pathways, subsequently promoting vascular endothelial growth factor (VEGF) expression and further inhibiting tight junction (TJ) expression,^[^ ^10^ ^]^ including the expression of claudins, occludins, and zonula occludens.^[^ ^11^ ^]^ The downregulation of these components directly impacts the structural integrity of the RPE monolayer and paracellular transport between vascular endothelial cells, allowing circulating immune cells to enter the RME and impairing immune privilege, representing a secondary BRB injury. The resulting inflammatory stimuli intensify RPE apoptosis, thus exacerbating primary BRB injury and establishing a detrimental feedback loop to disturb the RME. Therefore, a single intervention aimed at primary or secondary BRB injury cannot completely block disease progression.

Extensive investigations have revealed that the inflammasome, a multiprotein molecular platform assembled in response to various pathogen‐ and danger‐associated molecular patterns (PAMPs and DAMPs), plays an important role in the progression of DR.^[^ ^12^ ^]^ Inflammasomes are multimeric protein complexes initiated by nucleotide‐binding domain and leucine‐rich repeat receptors (NLRs) or absent in melanoma 2‐like receptors (ALRs).^[^ ^13^ ^]^ Among the NLR and ALR family members that form inflammasome complexes, NLR family, pyrin domain containing 3 (NLRP3) has been extensively studied in ocular pathologies. Prolonged activation of NLRP3 in RPEs induces pyroptosis, contributing to the abnormal activation of immune cells, dysregulated cytokine expression, and activation of compensatory angiogenic pathways. These events ultimately lead to retinal neuron apoptosis and visual impairment.^[^ ^14^ ^]^ NLRP3 inflammasome activation often requires priming signals mediated by reactive oxygen species (ROS).^[^ ^15^, ^16^ ^]^ As a result, inhibiting ROS generation in RPEs has a beneficial effect on modulating the RME by inhibiting high oxidative stress levels and reducing pyroptosis.

In addition to pyroptosis, sustained NLRP3 inflammasome activation also induces RPE dysfunction by upregulating the secretion and maturation of interleukin‐18 (IL‐18) through the engagement of NLRs and caspase‐1.^[^ ^17^ ^]^ Elevated ROS production not only primes NLRP3 but also increases pro‐IL‐18 transcription and expression.^[^ ^15^, ^18^ ^]^ Upon binding with IL‐18, the cytoplasmic portion of the IL‐18 receptor, which contains a Toll/IL‐1 receptor (TIR) domain, interacts with the TIR domain of myeloid differentiation factor 88 (MyD88).^[^ ^19^ ^]^ As a critical canonical adaptor for the Toll‐like receptor (TLR) family, MyD88 activates the NF‐κB and MAPK signaling pathways in RPEs, leading to the production of VEGF.^[^ ^20^ ^]^ Excessive VEGF secretion significantly increases BRB permeability by disrupting TJs, ultimately resulting in macular edema.^[^ ^21^ ^]^ Therefore, silencing *Myd88* can effectively block secondary BRB injury to subsequently remodel the RME by regulating the connexin between epithelial/endothelial cells and the extracellular matrix.

**Figure 1:**
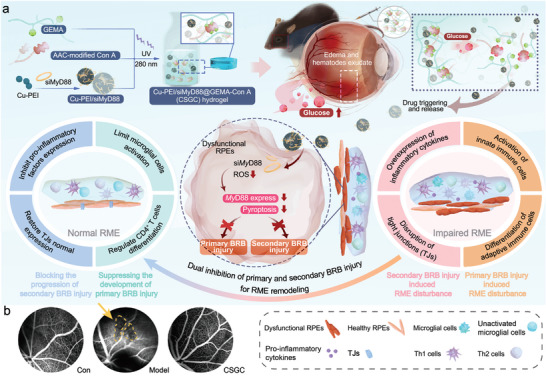
a) Schematic illustration showing that the CSGC hydrogel inhibits primary and secondary BRB injury to promote retinal microenvironment remodeling in DR. b) Preliminary fluorescent fundus angiography assay of CSGC hydrogel‐treated diabetic C57BL/6J mice with DR. The yellow arrow and boxes with dotted lines underscore the low‐contrast region induced by distal microvascular leakage.

In this study, by combining the silencing of *Myd88* with a reduction in oxidative stress, we proposed a dual inhibitory strategy targeting primary and secondary BRB injury to remodel the RME to attenuate DR deterioration (**Figure** **1**a). First, we developed a hydrogel based on glucosyloxyethyl methacrylate (GEMA)‐copolymerized concanavalin A (Con A) further used to encapsulate Cu‐polyethylenimine (PEI)/siMyD88 (a small interfering RNA targeting mouse *Myd88*‐loaded Cu‐PEI ultra small nanoparticles (USNPs)) (the final formed hydrogel is referred to as CSGC). This hydrogel, which responds to glucose stimuli, plays a crucial role in silencing MyD88 expression in RPEs. When plasma leakage occurs, the local glucose concentration in the vitreous body becomes similar to the blood glucose concentration, which results in the dissociation of the complex between Con A and pendant glucose in the hydrogel networks, leading to a decrease in cross‐linking density, swelling of the hydrogels, and subsequent release of Cu‐PEI/siMyD88 nanoparticles (NPs). Preliminary experiments demonstrated that the CSGC hydrogel effectively inhibited edema and distal vascular leakage in diabetic C57BL/6 mice (Figure 1b). Further pharmacological and mechanistic studies revealed that internalized Cu‐PEI USNPs act as antioxidants (mimicking catalase (CAT) and superoxide dismutase (SOD) activities), efficiently scavenging ROS in RPEs. ROS inhibition reduces RPE pyroptosis and the production of mature IL‐18. The inhibition of pyroptosis decreases microglial activation and differentiation of adaptive immune cells, thereby blocking primary BRB injury. Simultaneously, siMyD88, which is released from the endosome with the assistance of the positively charged counterpart of Cu‐PEI USNPs, caused RNA‐induced silencing complex ‐mediated cleavage of MyD88‐encoding mRNA, thereby inhibiting MyD88 expression. Combined with the decreased production of mature IL‐18, MAPK expression levels synergistically decreased, ultimately blocking secondary BRB injury by reducing VEGF and enhancing TJs expression. This study demonstrated the successful use of the CSGC hydrogel for RME remodeling through dual inhibition of primary and secondary BRB injury, representing a promising strategy for advanced therapy for the prevention and treatment of DR.

## 2 Results

### 2.1 Preparation and Characterization of Cu‐PEI USNPs

**Figure 2:**
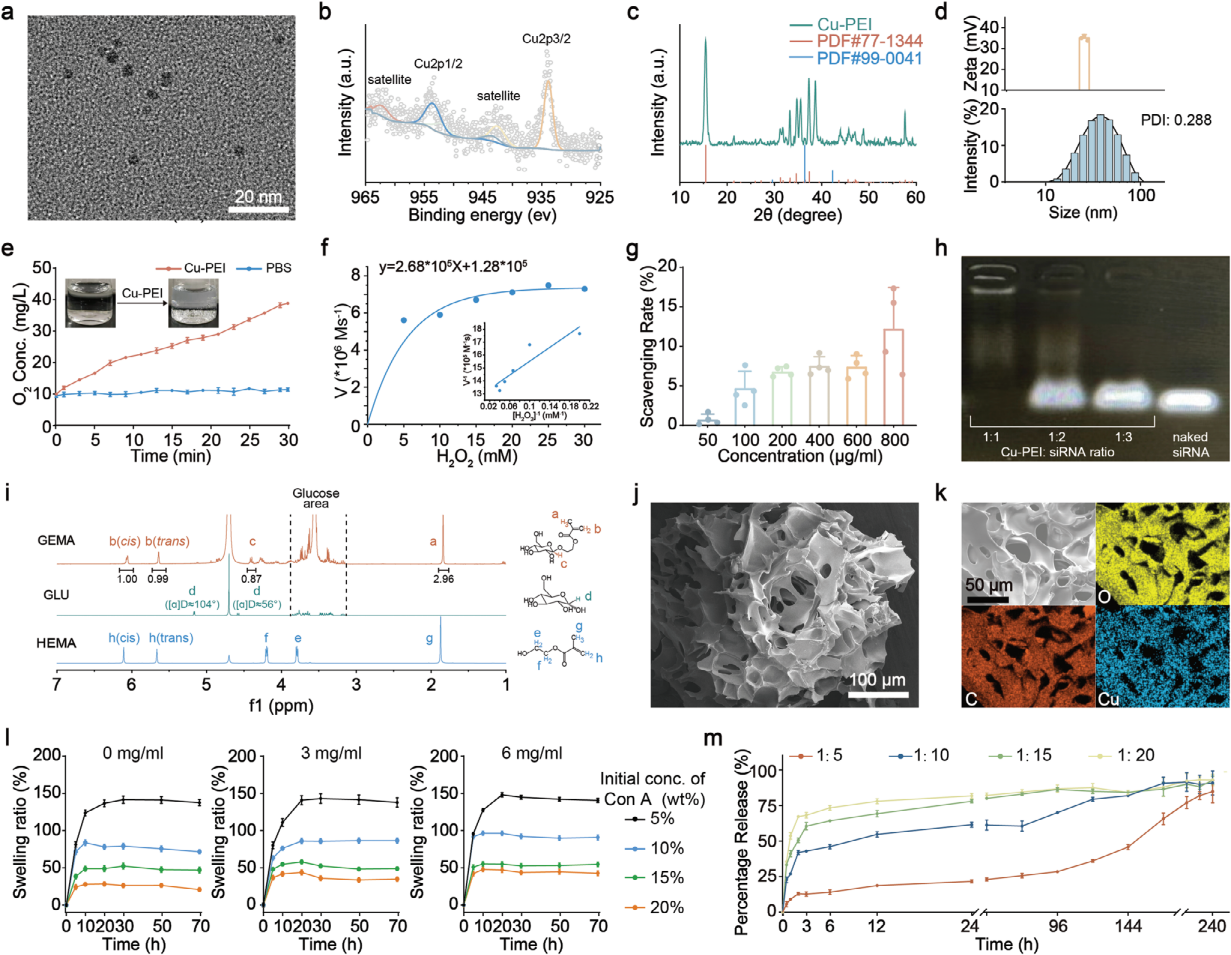
Physicochemical and functional characterization of the Cu‐PEI USNPs, GEMA, and CSGC hydrogels. a) TEM image of Cu‐PEI USNPs; scale bar: 20 nm. b) XPS spectra of Cu 2p. c) XRD analysis of the Cu‐PEI USNPs. d) Size and zeta potential of Cu‐PEI USNPs measured using DLS (*n* = 3). (e) O_2_ generation upon the addition of Cu‐PEI USNPs (200 µg mL^−1^) and PBS in H_2_O_2_ (50 mm) (*n* = 3). f) Steady‐state kinetic assay and Lineweaver–Burk plot for Cu‐PEI USNPs. g) Activity of Cu‐PEI USNPs in terms of the scavenging rate of ·O2^−^, as measured using a SOD kit (*n* = 4). h) Gel electrophoretic retardation analysis of the siRNA binding of Cu‐PEI USNPs. i) ^1^H‐NMR (400 MHz) spectra and peak assignments of GEMA, GLU, and HEMA within 7.0−1.0 ppm. j) SEM image of the CSGC hydrogel; scale bars: 100 µm. k) Elemental mapping of C, Cu, and O in the CSGC hydrogel; scale bars: 50 µm. l) The swelling percentage of the CSGC hydrogels in 0, 3, and 6 mg mL^−1^ glucose buffer (*n* = 3). m) Time course of siRNA release from hydrogels with different mass ratios of CSGC hydrogels in glucose buffer (*n* = 3). All the data are shown as the means ± SDs.

Prior to formulating the Cu‐PEI/MyD88 NPs and CSGC hydrogel, Cu‐PEI USNPs were synthesized using a one‐step rapid method (Figure S1a, Supporting Information), representing a pioneering approach. Transmission electron microscopy (TEM) images showed that the Cu‐PEI USNPs exhibited a uniform size distribution with an approximate diameter of 2 nm (**Figure** **2**a). The average hydrodynamic diameter of the Cu‐PEI USNPs, as measured using dynamic light scattering, was ≈55 nm (polydispersity index (PDI): 2.88), likely attributed to the strong positive charge (≈+35) on their surface, which facilitated the attraction of additional water molecules (Figure 2d). Subsequently, negatively charged siMyD88 was loaded onto the positively charged surface of the Cu‐PEI USNPs using electrostatic adsorption. As shown in Figure S1b (Supporting Information), the hydrodynamic diameter of the Cu‐PEI/siMyD88 NPs was ≈80 nm, with an average ζ potential of −6.51 mV. Then, we investigated the stability of the prepared Cu‐PEI USNPs and Cu‐PEI/siMyD88 NPs in DMEM (containing 10% FBS). After 10 days of incubation at 37 °C, the Cu‐PEI USNP and Cu‐PEI/siMyD88 NP solutions remained clear with slight morphological alterations, suggesting good dispersion and stability (Figure S1c, Supporting Information). X‐ray photoelectron spectroscopy (XPS) analysis revealed the presence of a distinct Cu^2+^ satellite peak at ≈962 eV, without any observable shift in the Cu2p3/2 peak (Figure 2b). This observation, which is consistent with the established copper X‐ray photoelectron spectra, suggested that the Cu‐PEI USNPs exhibited an oxidation state encompassing both Cu(I) oxide and Cu(II) oxide. Additionally, X‐ray diffraction (XRD) patterns (Figure 2c) indicated that the Cu‐PEI USNPs were composed of a composite of Cu^+^ and Cu^2+^ species, specifically Cu_2_O (PDF#99‐0041) and Cu_4_Cl_2_(OH)_6_ (PDF#77‐1344), further supporting the XPS analysis results.

To assess the CAT‐like activity of Cu‐PEI USNPs (Figure S1d, Supporting Information), multiple analytical methods, including electron spin resonance (ESR) spectroscopy, O_2_ generation, and H_2_O_2_ scavenging activity, were employed. As depicted in Figure S1e (Supporting Information), a progressive decrease in H_2_O_2_ concentration with increasing Cu‐PEI USNPs was observed. This enzymatic function resembling that of CAT facilitated the conversion of H_2_O_2_ into O_2_, thereby demonstrating the efficient time‐dependent degradation of H_2_O_2_ by Cu‐PEI USNPs (Figure 2e). Additionally, different H_2_O_2_ concentrations (ranging from 0 to 30 mm) were introduced into a solution containing Cu‐PEI USNPs (at a final concentration of 200 µg mL^−1^), and the rate of H_2_O_2_ consumption corresponding to each concentration was quantified. Based on the Beer–Lambert law, a curve relating the H_2_O_2_ concentration (horizontal axis) to the reaction rate (vertical axis) was plotted and fitted to the curve using the Michaelis–Menten saturation model (Figure 2f). Consequently, the initial reaction rates of H_2_O_2_ decomposition were calculated, and the *K_M_* and V_max_ values were 2.083 mm and 7.78 × 10^−6^ m s^−1^, respectively, for the Cu‐PEI USNPs. These determinations were made utilizing the double reciprocal plot of the Michaelis–Menten equation.

In addition to exhibiting CAT‐like activity, Cu‐PEI USNPs also demonstrated SOD‐like metalloenzyme activity, facilitating the dismutation of superoxide radicals (⋅O_2_^−^) into hydrogen peroxide (H_2_O_2_), as depicted in Figure S1f (Supporting Information). To evaluate the scavenging activity of ⋅O_2_^−^, an ESR test was initially conducted using 5‐tert‐butoxycarbonyl 5‐methyl‐1‐pyrroline N‐oxide (BMPO) as a probe. The results revealed a gradual decrease in the ⋅O_2_^−^ concentration as its initial concentration increased (Figure S1g, Supporting Information). Subsequently, the ⋅O_2_^−^ inhibition rate was quantified using a SOD assay kit (Figure 2g). Xanthine oxidase induces the oxidation of xanthine and generates ⋅O_2_^−^, which subsequently reduces a tetrazolium salt to produce a colored formazan product (nitroblue‐tetrazolium (NBT)‐formazan) that absorbs light. Therefore, the ⋅O_2_^−^ scavenging capacity of Cu‐PEI USNPs was assessed by measuring the degradation of NBT‐formazan utilizing NBT as the substrate. The results showed that the ⋅O_2_^−^ inhibition rate of Cu‐PEI USNPs (at a concentration of 200 mg mL^−1^) was 7.5% based on the absorbance of NBT‐formazan after a 30‐min reaction. These observations strongly support the application of Cu‐PEI USNPs as valuable nanozymes that provide defense against oxidative damage through CAT and SOD‐like activities.

### 2.2 Binding Affinity of siMyD88 to Cu‐PEI USNPs

In our pursuit of maximizing the loading efficiency of siMyD88, we identified the optimal weight ratio for Cu‐PEI USNPs and siMyD88. The strong positive charge of the Cu‐PEI USNPs facilitated the efficient condensation of negatively charged siRNA through electrostatic adsorption, thereby enhancing the siRNA loading efficiency. To minimize the potential elimination of free siMyD88 within the bloodstream, an agarose gel retardation assay (Figure 2h) was conducted to evaluate the binding affinity of Cu‐PEI USNPs to siMyD88 at different weight ratios. Remarkably, our results demonstrated that complete siRNA binding was achieved at a mass ratio of 1:1, whereas other weight ratios did not exhibit the same level of binding affinity. This finding highlights the superiority of the 1:1 ratio in terms of siRNA loading efficiency onto USNPs. To achieve a high silencing level in subsequent in vitro and in vivo experiments, we selected a 1:1 weight ratio of Cu‐PEI USNPs and siMyD88 as the optimal ratio.

### 2.3 GEMA Synthesis and Characterization

In a separate step, we synthesized GEMA for the subsequent creation of the CSGC hydrogel (Figure S2a, Supporting Information). For the synthesis process, we used d‐glucose and hydroxyethyl methacrylate (HEMA) as raw materials, and *β*‐glucosidase was employed as a catalyst. We performed a comprehensive structural characterization of the resulting GEMA compound. First, Fourier transform infrared (FTIR) spectroscopy was utilized to qualitatively analyze GEMA, HEMA, and d‐glucose (Figure S2b, Supporting Information). Compared to d‐glucose, a distinctive characteristic band of GEMA appeared at ≈1715 cm^−1^, corresponding to the methacrylate carbonyl groups (C═O stretch). Furthermore, specific asymmetric and symmetric stretching vibrations of carbon‒hydrogen (ν_as_CH and ν_s_CH) derived from glucose were observed at ≈2700 and 2790 cm^−1^, respectively, for GEMA but not for HEMA. Additionally, the ^1^H‐nuclear magnetic resonance (^1^H‐NMR) spectrum showed that the peak integration ratio of the protons (H_b_) bonded to the carbon of an alkene double bond and a proton (H_c_) bonded to the carbon, which is bonded to two oxygens of the pyranoid ring, was close to 1:1 (Figure 2i). The glucose region was clearly discernible within the range of 3.2–4.2 ppm. Notably, a downfield shift was observed for both the *α*‐ and *β*‐hydroxyl methylene protons, from 3.79 to 4.07 ppm for He and from 4.20 to 4.28 for Hf. The FTIR and ^1^H‐NMR results confirmed successful GEMA synthesis.

### 2.4 Preparation of CSGC Hydrogels and Analysis of Glucose‐Responsive Behavior

Glucose‐responsive hydrogels have gained considerable attention as intelligent materials in the biochemical and biomedical fields due to their ability to autonomously sense environmental glucose concentrations and trigger structural transformations. Our study focused on the development of a glucose‐responsive hydrogel specifically designed for the treatment of DR. One of the key components of the hydrogel is Con A, a natural lectin protein originally derived from the jack bean that possesses four binding sites for glucose.^[^ ^22^ ^]^ Additionally, GEMA, which contains a pendant glucose moiety, copolymerizes with acrylic acid (AAc)‐modified Con A to form a densely cross‐linked network structure. In this work, AAc‐modified Con A was prepared using a standard 1‐ethyl‐3‐(3‐dimethylamino propyl) carbodiimide hydrochloride (EDC) catalytic reaction (Figure S2c, Supporting Information). The uniform and interconnected morphology of the CSGC hydrogel was observed using scanning electron microscopy (SEM) as depicted in Figure 2j. Furthermore, elemental mapping and ultraviolet (UV)−visible (*vis)* spectroscopy were conducted to provide compelling evidence confirming the successful loading of Cu‐PEI USNPs and the synthesis of the CSGC hydrogel (Figure 2k; Figure S2d, Supporting Information).

Exploring glucose‐responsive behavior is crucial for comprehending the dynamics of drug release under pathological conditions. Generally, the swelling ability of hydrogels affects their responsive behavior, and the swelling behavior is strongly influenced by the crosslinking density.^[^ ^23^ ^]^ In our study, AAc‐modified Con A played a pivotal role as a cross‐linking agent. Therefore, it was crucial to determine the actual concentration of the immobilized cross‐linking agent in the CSGC hydrogel to investigate its glucose‐responsive behavior. Figure S2e (Supporting Information) shows a good linear correlation between the amount of Con A immobilized in the hydrogel and the initial amount of Con A. Specifically, ≈81.5% of the AAc‐modified Con A was copolymerized with GEMA during the preparation of the CSGC hydrogel.

Next, we examined the influence of different initial concentrations of Con A on the swelling ratio of the CSGC hydrogels. The results indicated that the swelling ratio gradually increased with a decreasing initial concentration of AAc‐modified Con A in the 0, 3, and 6 mg mL^−1^ glucose buffer solutions (Figure 2l). However, no significant change in the swelling ratio was noted for hydrogels with an initial 5% Con A concentration in buffers with different glucose concentrations. In contrast, the swelling ratio for the hydrogel with an initial 20% Con A concentration in 6 mg mL^−1^ glucose buffer was ≈1.33 times greater than that in 3 mg mL^−1^ glucose buffer, and ≈1.69 times greater than that in 0 mg mL^−1^ glucose buffer. This finding indicated that this type of hydrogel exhibited a sensitive swelling ability in response to fluctuations in the environmental glucose concentration. Moreover, the swelling ratio of the hydrogel containing an initial 20% Con A concentration was much lower than that of the other types of hydrogels at the same glucose concentration, which was expected to maintain normal intraocular pressure in subsequent experiments. Therefore, we selected an initial concentration of 20% Con A as the optimal formulation parameter for the CSGC hydrogel in subsequent experiments.

Next, we proceeded with the glucose‐responsive analysis of the hydrogel containing an initial 20% Con A concentration. A RiboGreen assay was conducted to investigate the release of siRNA from the CSGC hydrogel in glucose buffer. We observed that the hydrogel with an initial 20% Con A concentration reached complete swelling at ≈10 h in glucose buffer (Figure 2l). Simultaneously, the hydrogels with a mass ratio of 1:10, 1:15, and 1:20 of siRNA to Con A‐GEMA released ≈70–75% of the siRNA within 12 h in a buffer with 3 mg mL^−1^ glucose (Figure 2m). These findings indicated that these three loading configurations result in the release of most of the siRNA during the responsive swelling stage. In contrast, the hydrogel with a mass ratio of 1:5 released 75% siRNA after ≈240 h, demonstrating excellent capability for meeting the requirements of long‐term siRNA release in ophthalmic preparations. Consequently, a CSGC hydrogel loaded with a ≈16.7% mass fraction of siMyD88 was used for subsequent investigations.

### 2.5 Injectability, Stability, and Degradability of CSGC Hydrogel

A series of studies have been conducted to examine the shear, viscosity, and degradation properties of the optimized CSGC hydrogels. In the shear and viscosity variation assessment, we found that the sample exhibited shear thinning characteristics and moderate viscosity (Figure S2f, Supporting Information), implying homogeneous dispersity after injection into the eyes. Moreover, the CSGC hydrogel showed excellent injectability when it was injected effortlessly through a 27‐gauge needle syringe (inset in Figure S2f, Supporting Information). Additionally, dynamic mechanical analysis (DMA) was performed within a strain range spanning from −120% to 0% to elucidate the mechanical properties of the CSGC hydrogel. The values of the storage modulus (E´) and loss modulus (Eʺ) remained consistently stable throughout the entire strain range, demonstrating that it had good mechanical torsion ability (Figure S2g, Supporting Information). As previously mentioned, given that ≈81.5% of AAc‐modified Con A copolymerized with GEMA in the GEMA‐copolymerized Con A hydrogel, the crosslinking density of the CSGC hydrogel was greater than that of many other types of hydrogels. Therefore, it can be inferred that the CSGC hydrogel possesses favorable stability properties. As expected, even in buffer at a pH of 5.5 for 10 days, the degradation ratio of the CSGC hydrogel remained lower than 30%, confirming its great stability (Figure S2h, Supporting Information). Given the application environment of the CSGC hydrogel, artificial tears were used as controls. Interestingly, the degradation ratio of the CSGC hydrogel in the artificial tears was obviously greater than that in the buffer at a pH of 5.5, emphasizing the potential application of CSGC the hydrogel in eye disease treatment.

### 2.6 In Vitro Transfection Efficiency and Cytotoxicity of Cu‐PEI/siMyD88 NPs

**Figure 3:**
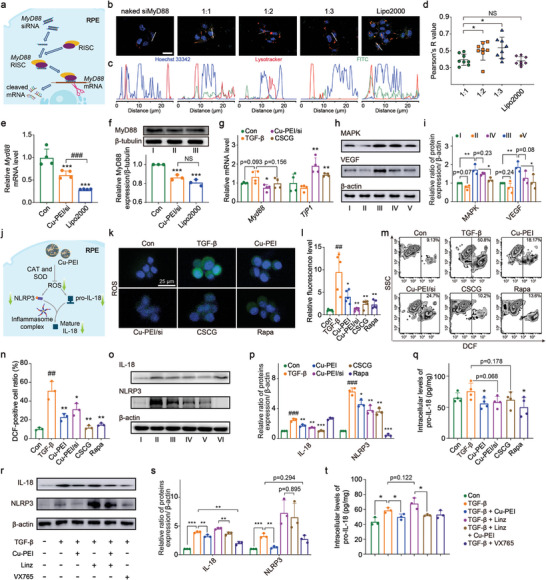
The CSGC hydrogel mediated MyD88 silencing, ROS scavenging, and inflammasome inhibition in vitro. a) Schematic illustration of the transfection of siMyD88 and the inhibition of MyD88 expression in RPEs. b) Confocal microscopy images showing the efficiency of internalization and endosomal escape of Cu‐PEI/FITC‐labeled siMyD88 NPs after treatment with different weight ratios of Cu‐PEI and siRNA in RPEs for 6 h (green: FITC‐labeled siMyD88; red: LysoTracker; blue: DAPI); scale bars: 25 µm. The corresponding c) fluorescence curve analyses and d) Pearson's R‐value analysis (*n* = 8). e) RT‒qPCR analysis of the *Myd88* mRNA level in RPEs after treatment with Cu‐PEI/siMyD88 NPs (Cu‐PEI/SI) and Lipo2000 (*n* = 4). ^***^ *p* < 0.001, test group versus Con group; ^###^ *p* < 0.001, Lipo2000 group versus Cu‐PEI/siMyD88 group. f) Western blotting and quantitative analysis of MyD88 expression in RPEs after treatment with Cu‐PEI/siMyD88 NPs (Cu‐PEI/SI) and Lipo2000. I: Control, II: Cu‐PEI/siMyD88 NPs, III: Lipo2000/siMyD88 (*n* = 3). ^*^ *p* < 0.05, test group versus Con group. ^#^ *p* < 0.05, Lipo2000 group versus Cu‐PEI/siMyD88 group. g) RT‒qPCR of *Myd88* and *Tjp1* mRNA levels in RPEs after treatment with TGF‐*β* (10 ng mL^−1^) and Cu‐PEI/siMyD88 NPs or CSGC hydrogel (*n* = 4). ^*^ *p* < 0.05, ^**^ *p* < 0.01, test group versus TGF‐*β* group. h) Western blotting and i) quantitative analysis of MAPK and VEGF protein expression levels in RPEs after exposure to different treatments. I: Control, II: Control + Cu‐PEI USNPs, III: TGF‐*β*, IV: TGF‐*β* + Cu‐PEI USNPs, V: TGF‐*β* + Cu‐PEI/siMyD88 NPs (*n* = 3). ^*^ *p* < 0.05, ^**^ *p* < 0.01. j) Schematic illustration of the ability of the CSGC hydrogel to scavenge ROS and inhibit inflammasome activity in RPEs. k) CLSM micrographs of total intracellular ROS in RPEs after different treatments (scale bar: 25 µm) and l) related quantitative analysis (*n* = 5). ^*^ *p* < 0.05, ^**^ *p* < 0.01, test group versus TGF‐*β* group; ^##^ *p* < 0.01, TGF‐*β* group versus Con group. m) A flow cytometry assay with DCFH‐DA was performed to detect the movement of ROS in RPEs after different treatments, and n) a related analysis (*n* = 3). ^*^ *p* < 0.05, ^**^ *p* < 0.01, test group versus TGF‐*β* group; ^##^ *p* < 0.01, TGF‐*β* group versus Con group. o) Western blotting and p) related quantitative analysis of NLRP3 and IL‐18 protein expression levels in RPEs after different treatments. I: Control, II: TGF‐*β*, III: Cu‐PEI USNPs, IV: Cu‐PEI/siMyD88 NPs, V: CSGC hydrogel, and VI: Rapa (*n* = 3). ^*^ *p* < 0.05, ^**^ *p* < 0.01, ^***^ *p* < 0.001, test group versus TGF‐*β* group; ^###^ *p* < 0.001, TGF‐*β* group versus Con group. q) Intracellular pro‐IL‐18 levels in RPEs after exposure to different treatments were detected using ELISA kits (*n* = 4). ^*^ *p* < 0.05, test group versus TGF‐*β* group. r) Western blotting and s) related quantitative analysis of IL‐18 and NLRP3 protein expression levels in RPEs in the presence or absence of linezolid, an NLRP3 agonist (*n* = 3). ^**^ *p* < 0.01, ^***^ *p* < 0.001. t) Intracellular pro‐IL‐18 levels in RPEs in the presence and absence of linezolid (*n* = 3). ^*^ *p* < 0.05. All the data are presented as the means ± SDs, and statistical analysis was performed using one‐way ANOVA.

Prior to the initial pharmacological investigations, a series of experiments were conducted to determine the optimal weight ratio between siMyD88 and Cu‐PEI USNPs to maximize transfection efficiency. The process of siMyD88 transfection and RNA interference is illustrated in **Figure** **3**a. Initially, the transfection efficiency of different formulations was assessed by measuring the fluorescence intensity of internalized fluorescein isothiocyanate (FITC)‐modified siMyD88 and lysosomes labeled with LysoTracker Red. RPEs were transfected with various mass ratios of siMyD88:Cu‐PEI, ranging from 1:1 to 1:3, whereas naked siMyD88 and Lipofectamine 2000 (Lipo2000) were used as controls. Figure S3a (Supporting Information) shows comparable LysoTracker Red signals in the 1:1, 1:2, and 1:3 groups, confirming the efficient endocytosis of Cu‐PEI/siMyD88 NPs by RPEs after 4 h of incubation, which was observed in lysosomes. Furthermore, a minimal FITC signal was observed in the naked siMyD88 group, whereas the FITC intensity increased proportionally with a decreasing siMyD88:Cu‐PEI ratio (Figure 3b; Figure S3a, Supporting Information). Combined with the results of the fluorescence curve evaluation (Figure 3c) and Pearson's R‐value analysis (Figure 3d), these findings suggested that Cu‐PEI/siMyD88 NPs successfully inhibited lysosomal storage, exhibiting an approximately mass ratio‐dependent pattern. Consequently, a mass ratio of 1:1 was chosen as the optimal for the components of Cu‐PEI/siMyD88 NPs. Then, MyD88 expression was examined using real time (RT)‐quantitative polymerase chain reaction (qPCR) and Western blotting (*β*‐tubulin expression was chosen as an internal control). The results showed that Cu‐PEI/siMyD88 NPs significantly reduced the levels of *Myd88* mRNA and MyD88 protein in RPEs (Figure 3e,f). Interestingly, although the *Myd88* mRNA level in the Lipo2000 group was 55.1% lower than that in the Cu‐PEI/siMyD88 group, the overall expression level decreased by only 6.2%. This marginal decrease may be due to negative feedback in the process by which MyD88 participates in TLR immune regulation to ensure cellular integrity and prevent apoptosis.

The cytotoxicity of delivery vehicles is a critical factor to consider. Therefore, we conducted a comprehensive assessment of cell viability after treating RPEs with Cu‐PEI USNPs, Lipo2000, and PEI, both in the presence and absence of siMyD88. RPE viability assays demonstrated that the cytotoxicity induced by Cu‐PEI USNPs on RPEs was significantly lower than that induced by Lipo2000 and PEI, regardless of the presence of siMyD88 (Figure S3b, Supporting Information). By integrating the results of the lactate dehydrogenase (LDH) release activity assay (Figure S3c, Supporting Information), which evaluates cell membrane integrity following exposure to Cu‐PEI USNPs, Lipo2000, and pure PEI, a noteworthy observation emerged. The strong transfection ability of Lipo2000 significantly triggered apoptosis and membrane rupture in RPEs. Pure PEI exhibited a similar, albeit slightly less potent, effect than Lipo2000. In contrast, Cu‐PEI USNPs had minimal impact, as evidenced by marginal apoptosis and cell membrane rupture, which had limited statistical significance. These findings strongly indicate that neither Cu‐PEI USNPs nor Cu‐PEI/siMyD88 induced apoptosis or necrosis in RPEs.

### 2.7 CSGC Hydrogel Activates TJs Expression by Inhibiting NLRP3 and IL‐18 Maturation

The intricate regulation of TJs has a profound impact on DR development and progression. Previous investigations have revealed the obvious suppressive effect of transforming growth factor (TGF)‐*β*1 treatment on the expression of zonula occludens (ZO)‐1, a critical TJ protein, in RPEs.^[^ ^24^ ^]^ Therefore, we considered TGF‐*β*1 as an ideal modeling agent for conducting in vitro pharmacological and mechanistic studies on RPEs. Initially, we evaluated the cytotoxicity of the CSGC hydrogel on RPEs and found no apparent cytotoxic effects at varying concentrations, as shown in Figure S3d (Supporting Information). Subsequently, RPEs preincubated with Cu‐PEI/siMyD88 NPs or CSGC hydrogels were treated with serum‐free DMEM containing TGF‐*β*1 (10 ng mL^−1^) for 12 h, followed by qRT‒PCR analysis (Figure 3g). The results revealed a 32% increase in *Myd88* mRNA levels and a 26% decrease in *Tjp1* mRNA levels in the TGF‐*β* group compared to the control group, with both P values approaching 0.05. Compared with the TGF‐*β* group, the Cu‐PEI/siMyD88 NP group exhibited a 44% decrease in *Myd88* mRNA levels and a 158% increase in *Tjp1* mRNA levels, indicating that Cu‐PEI/siMyD88 NPs significantly enhanced ZO‐1 expression in RPEs. Furthermore, the alteration in *Tjp1* mRNA levels in the Cu‐PEI/siMyD88 group was approximately fourfold greater than that observed for *Myd88* mRNA, suggesting that ZO‐1 regulation may not be solely attributed to the silencing of *Myd88* but likely involves a cascade effect synergistically induced by Cu‐PEI USNPs in combination with *Myd88* silencing. Interestingly, the efficacy of the CSGC hydrogel appeared to be weaker than that of the Cu‐PEI/si group, possibly due to the incomplete release of the Cu‐PEI/siMyD88 NPs resulting from the sustained release of the CSGC hydrogel during preincubation. Notably, qRT‒PCR results showed that changes in *Tjp1* mRNA levels in the CSGC group were still approximately four times greater than that in the *Myd88* mRNA levels.

Subsequently, we performed a systematic investigation of protein alterations within the pathway upstream of ZO‐1. Previous studies have confirmed that VEGF expression upregulates TJs in RPEs.^[^ ^25^ ^]^ Moreover, inhibition of MyD88 influences the NF‐kB and MAPK signaling pathways in various cell types.^[^ ^26^ ^]^ Therefore, we first explored the effects of *Myd88* silencing on MAPK and VEGF expression. Western blotting and quantitative analysis provided evidence that Cu‐PEI/siMyD88 NPs effectively reduced MAPK and VEGF expression in TGF‐*β*‐activated RPEs (Figure 3h,i). Notably, treatment with Cu‐PEI USNPs alone resulted in slight decreases in MAPK and VEGF expression, with limited statistical significance, both in normal RPEs and TGF‐*β*‐activated RPEs. The ability of VEGF to compromise BRB integrity by acting directly on the TJs between RPEs was demonstrated,^[^ ^27^ ^]^ further emphasizing the synergistic effect of Cu‐PEI USNPs and *Myd88* silencing on the ZO‐1‐related pathway. We also explored the mechanism by which Cu‐PEI USNPs affect the MAPK/VEGF pathway. We hypothesized that Cu‐PEI USNPs hindered MAPK and VEGF expression by scavenging ROS. In subsequent studies, rapamycin (Rapa) was chosen as a positive control due to its strong inhibitory effect on VEGF production and angiogenesis‐related pathway blockade.^[^ ^28^ ^]^ To assess the diverse ROS‐scavenging capabilities of Cu‐PEI USNPs and CSGC hydrogels in vitro, we employed 2′,7′‐dichlorodihydrofluorescein diacetate (DCFH‐DA) as an H_2_O_2_ probe and dihydroethidium (DHE) as a probe for ·O_2_^−^. As shown in Figure S4a (Supporting Information), compared with TGF‐*β*‐activated RPEs, the levels of H_2_O_2_ (green fluorescence signals) and ·O_2_^−^ (red fluorescence signals) significantly decreased following incubation with the Cu‐PEI USNPs and CSGC hydrogels. Consistent with these observations, confocal laser scanning microscopy (CLSM) revealed robust green fluorescence in TGF‐*β*‐treated RPEs, indicating ROS accumulation (Figure 3k,l). Cu‐PEI USNPs, Cu‐PEI/siMyD88 NPs, and the CSGC hydrogel significantly inhibited ROS accumulation in RPEs. Furthermore, similar results were obtained using flow cytometry and corresponding quantitative analysis of total intracellular ROS in TGF‐*β*‐stimulated RPEs (Figure 3m,n).

Previous studies confirmed that ROS mediates MAPK pathway activation by promoting IL‐18 secretion in multiple diseases.^[^ ^29^ ^]^ Therefore, we examined the changes in mature IL‐18 levels in TGF‐*β*‐stimulated RPEs. The results revealed a significant decrease of ≈35% in mature IL‐18 in both the Cu‐PEI and Cu‐PEI/si groups (Figure 3o,p), suggesting that the CSGC hydrogel inhibits mature IL‐18 production by scavenging ROS, thereby reducing MAPK expression in RPEs. The maturation of IL‐18 involves *Il18* mRNA transcription and pro‐IL‐18 secretion. Consequently, we also assessed pro‐IL‐18 expression in TGF‐*β*‐activated RPEs. We observed a 26% reduction in pro‐IL‐18 in the Cu‐PEI group compared to the TGF group (Figure 3q), indicating that Cu‐PEI USNPs indeed partly suppressed pro‐IL‐18 expression by inhibiting ROS production. However, notably, although mature IL‐18 decreased by ≈60% in the CSGC group, pro‐IL‐18 decreased by only 20% to 25% in the Cu‐PEI, Cu‐PEI/si, and CSGC groups. This discrepancy suggests that an additional mechanism collaboratively hinders IL‐18 maturation. Considering the direct association between IL‐18 maturation and NLRP3 inflammasome activation in RPEs, we hypothesize that the synergistic mechanism likely involves the inhibition of NLRP3 inflammasome complex formation by Cu‐PEI USNPs. To support this hypothesis, we examined the expression of NLRP3 in TGF‐*β*‐stimulated RPEs. Quantitative analysis indicated a ≈55% reduction in NLRP3 expression in the CSGC group compared to the TGF‐*β* group (Figure 3p,q), providing further support for our hypothesis. The CSGC hydrogel effectively inhibited NLRP3 and pro‐IL‐18 expression by scavenging ROS in RPEs, thereby reducing mature IL‐18 levels.

To elucidate the specific role of the ROS scavenging activity of Cu‐PEI USNPs in inhibiting IL‐18 maturation and relevant pathways, linezolid and VX765 were used in this study. Linezolid (Linz), an oxazolidinone antibiotic, is an NLRP3 agonist that activates the NLRP3 inflammasome independently of ROS.^[^ ^30^ ^]^ VX765 is a caspase‐1 inhibitor that inhibits downstream inflammasome assembly and IL‐18 maturation.^[^ ^31^ ^]^ As depicted in Figure 3r–t, VX765 (50 µm, pretreatment for 2 h) did not significantly inhibit NLRP3 or pro‐IL‐18 expression but strongly suppressed IL‐18 maturation, indicating that the inflammasome plays a crucial role in IL‐18 maturation in RPEs. Linz (10 µg mL^−1^, 12 h) significantly enhanced NLRP3 expression in RPEs, and upon coexposure to Cu‐PEI USNPs, Cu‐PEI USNPs no longer reduced NLRP3 expression. Interestingly, even in the presence of Linz, Cu‐PEI USNPs still significantly reduced mature IL‐18 levels, suggesting that Cu‐PEI inhibited IL‐18 maturation through alternative pathways. Tarallo et al. reported that the ROS inhibitor diphenyliodonium blocks the upregulation of IL‐18 mRNA in RPEs.^[^ ^15^ ^]^ Furthermore, Cu‐PEI USNPs reduced pro‐IL‐18 levels regardless of the presence of Linz, confirming that Cu‐PEI USNPs also directly inhibit the generation of pro‐IL‐18.The schematic illustration in Figure 3j depicts the pathway influenced by Cu‐PEI USNPs, which involves the inhibition of NLRP3 expression and the regulation of mature IL‐18 production through ROS scavenging.

### 2.8 The CSGC Hydrogel Inhibited Apoptosis and Pyroptosis in RPEs

**Figure 4:**
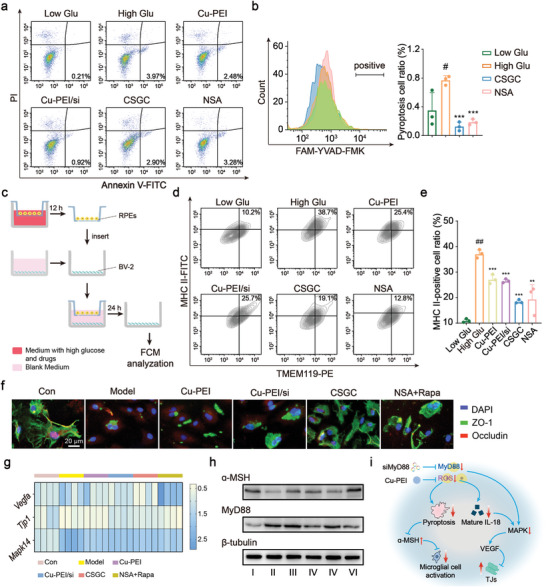
Blocking effects of the CSGC hydrogel on primary BRB injury and secondary BRB injury in vitro. a) Flow cytometry analysis of apoptotic RPE cells after different treatments. b) Flow cytometry assay of pyroptotic RPEs after different treatments and related quantitative analysis (*n* = 3). ^***^ *p* < 0.001, test group versus High Glu group; ^#^ *p* < 0.05, High Glu group versus Low Glu group. c) Experimental process illustrating how the CSGC hydrogel affected the antigen‐presenting ability of microglia. d) Flow cytometry analysis of the antigen‐presenting ability of microglia after different treatments and e) related quantitative analysis (*n* = 3). ^**^ *p* < 0.01, ^***^ *p* < 0.001, test group versus High Glu group; ^##^ *p* < 0.01, High Glu group versus Low Glu group. f) Immunofluorescence assay of occludin (red) and ZO‐1 (green) expression in RPE monolayers; scale bar: 20 µm. g) Heatmap showing the differences in the mRNA levels of key genes in RPEs after different treatments; *n* = 4 in each group. h) Western blotting analysis of *α*‐MSH and MyD88 protein expression levels in RPEs after exposure to different treatments. I: Con, II: Model, III: Cu‐PEI USNPs, IV: Cu‐PEI/siMyD88 NPs, V: CSGC hydrogel, and VI: NSA + Rapa. i) Schematic illustration of the remodeling of the RME by the CSGC hydrogel. All the data are presented as the means ± SDs, and statistical analysis was performed using one‐way ANOVA.

Given the demonstrated ability of CSGC to decrease NLRP3 and IL‐18 expression, it is crucial to investigate the impact of the CSGC hydrogel on pyroptosis. Previous studies have elucidated the dose‐dependent contribution of high glucose to pyroptosis in RPEs.^[^ ^32^ ^]^ Therefore, we investigated the anti‐apoptotic and anti‐pyroptotic effects of the CSGC hydrogel on RPEs using 50 mm glucose medium to model high‐glucose conditions. Here, the pyroptosis inhibitor necrosulfonamide (NSA) served as a positive control. As shown in **Figures** **4**a and S4b (Supporting Information), we observed a greater percentage of apoptotic RPE cells under high‐glucose conditions than under low‐glucose conditions. Both the Cu‐PEI/siMyD88 NPs and CSGC hydrogels significantly inhibited the ratio of viable apoptotic cells and nonviable apoptotic cells in RPEs under high glucose concentrations. Similarly, compared to those in the low‐glucose group, the percentage of pyroptotic RPEs increased by ≈100% in the high‐glucose group. The Cu‐PEI/siMyD88 NPs and CSGC hydrogels effectively reversed the pro‐pyroptotic effect of high glucose concentrations, and the efficacy was comparable to that of NSA (Figure 4b; Figure S4c,d, Supporting Information). These findings strongly indicate that the CSGC hydrogel protects RPEs against the high‐glucose microenvironment associated with diabetes.

### 2.9 The CSGC Hydrogel Inhibited Primary BRB Injury Induced Microglial Activation In Vitro

The occurrence of pyroptosis and apoptosis in RPEs directly affects the structural integrity of RPE monolayer cells in the retinal microenvironment, thereby influencing the permeability of the BRB and ocular immune privilege. Disruption of immune privilege leads to the migration of immune cells from the circulatory system to the eyes, exacerbating the inflammatory response in DR. *α*‐melanocyte‐stimulating hormone (MSH), a crucial negative regulator secreted by RPEs, plays a central role in maintaining immune homeostasis within the eye. Previous studies have reported a significant reduction in *α*‐MSH secretion when RPEs are impaired.^[^ ^33^ ^]^ Therefore, we initially evaluated *α*‐MSH expression in RPEs exposed to a high‐glucose environment. The results indicated a substantial decrease in *α*‐MSH expression in RPEs under high‐glucose conditions compared to low‐glucose conditions. Importantly, the Cu‐PEI USNPs, Cu‐PEI/siMyD88 NPs, and CSGC hydrogels significantly restored *α*‐MSH expression levels in RPEs (Figure 4e,h). Concurrently, we examined MyD88 expression in RPEs in a high‐glucose model. Cu‐PEI/siMyD88 NPs and CSGC hydrogels significantly reduced MyD88 expression in RPEs, whereas Cu‐PEI USNPs did not have the same effect. These results collectively suggest that the recovery of *α*‐MSH expression is primarily attributed to the inhibition of RPE pyroptosis by Cu‐PEI USNPs, independent of *Myd88* silencing.

To evaluate the remodeling effects of the CSGC hydrogel on the retinal microenvironment, we investigated the activation of innate immune cells within the retina. Microglia, a crucial component of the innate immune system in the retinal microenvironment, have been implicated in various eye diseases.^[^ ^34^ ^]^ Microglia are primarily distributed in the inner layer of the retina and, in their quiescent state, form a mononuclear phagocyte system with macrophages, serving immune surveillance and maintaining retinal microenvironment homeostasis. Upon changes in the microenvironment, resting microglia undergo activation, assuming roles in antigen presentation, cytokine release, inflammation, and process of tissue injury or repair.^[^ ^35^ ^]^ Earlier studies have highlighted the negative effects of *α*‐MSH on the differentiation of microglia toward the immune activation phenotype and subsequent mediated inflammation.^[^ ^33^ ^]^ Using transwell chambers to co‐culture high glucose‐activated RPEs with BV‐2 microglia (the operation process is outlined in Figure 4c), flow cytometry results demonstrated that impaired RPEs significantly increased microglial activation. In addition, the GC gel (CSGC hydrogel not loaded with Cu‐PEI/siMyD88 NPs) did not inhibit microglial activation (Figure S4f,g, Supporting Information). In contrast, pretreatment of RPEs with Cu‐PEI USNPs, Cu‐PEI/siMyD88 NPs, or the CSGC hydrogel significantly reduced the proportion of activated microglia (Figure 4d,e). Furthermore, RPEs pretreated with NSA also showed decreased microglial activation, indicating that the suppression of microglial cell activation in the microenvironment by the CSGC hydrogel primarily stems from its inhibition of RPE pyroptosis.

### 2.10 The CSGC Hydrogel Protected BRB Against Secondary Injury

To comprehensively validate the impact of the CSGC hydrogel on the extracellular matrix junction complex in the retinal microenvironment in these two in vitro cellular models and elucidate its inhibitory effects and mechanism on secondary BRB injury, we combined the two modeling methods. Initially, we examined the influence of the CSGC hydrogel on ZO‐1 and occludin expression in RPEs exposed to both TGF‐*β* and high‐glucose environments. As illustrated in Figure 4f, the CSGC hydrogel notably mitigated the decrease in ZO‐1 expression in this combined model. However, compared with that in the Model group, occludin expression did not significantly differ among the treatment groups, suggesting that the impact of the CSGC hydrogel on the extracellular matrix junction complex in the retinal microenvironment is predominantly manifested in ZO‐1 protein. Subsequently, we validated the regulatory mechanism of ZO‐1 expression. The heatmap (Figure 4g; Figure S4h, Supporting Information) illustrates that the CSGC hydrogel indeed restored ZO‐1 expression through the MAPK/VEGF pathway. Synthesizing all the outcomes from in vitro pharmacological and mechanistic studies, we summarized the mechanism of action of the CSGC hydrogel on RME remodeling through blocking primary BRB injury and preventing secondary BRB injury after the release of Cu‐PEI/siMyD88 NPs (Figure 4i).

### 2.11 The CSGC Hydrogel Inhibited DR Development In Vivo

**Figure 5:**
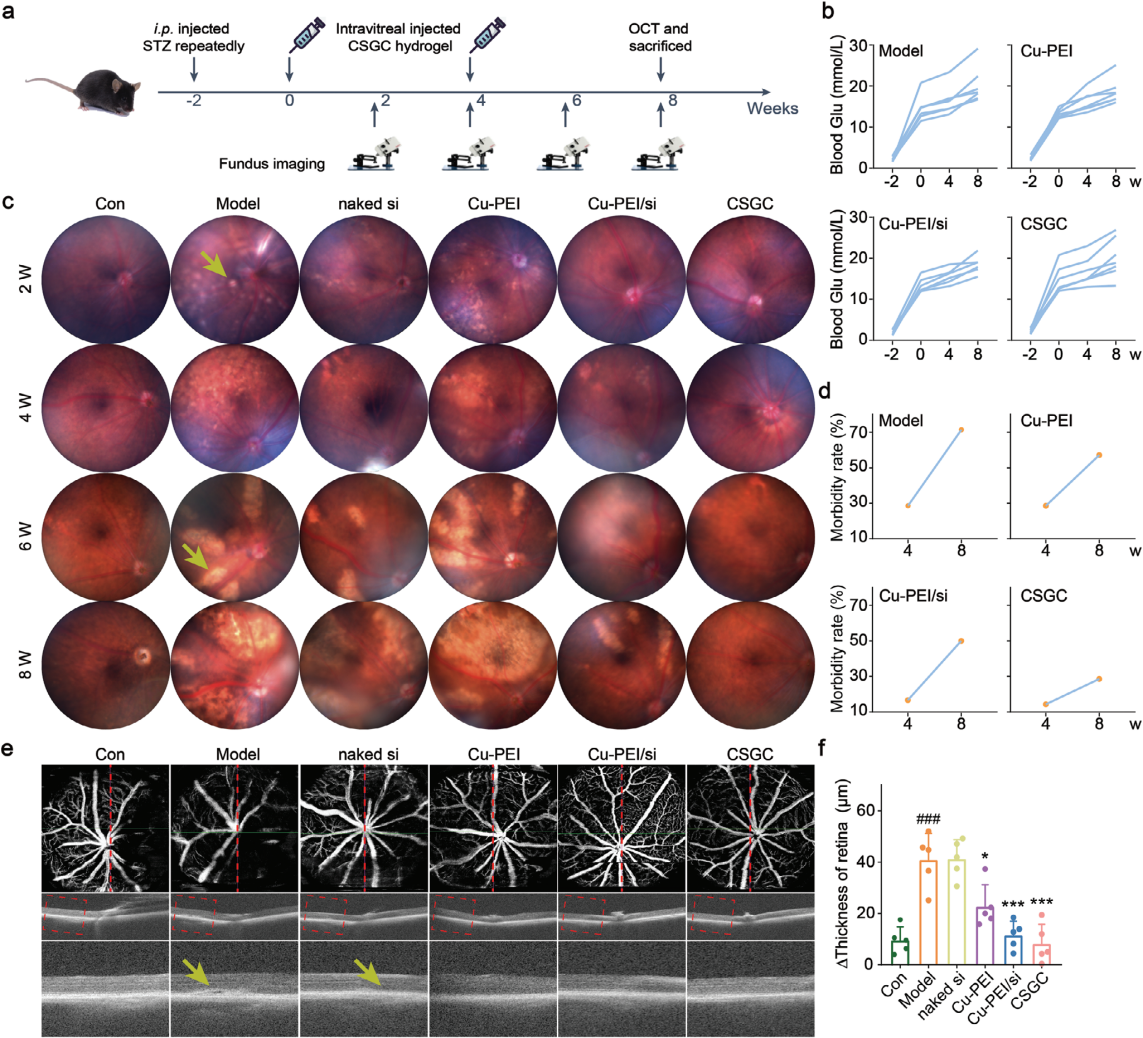
The CSGC hydrogel inhibited DR development in diabetic C57BL/6 mice. a) Schematic illustration of the animal treatment protocols. b) Changes in blood glucose levels in diabetic C57BL/6J mice at −2 to 8 weeks in each group (*n* = 7 for Model, Cu‐PEI and CSGC groups; *n* = 6 for Cu‐PEI/si group). c) Representative photographs of retinal images of C57BL/6J mice after exposure to different treatments. The degree of diabetic edema is indicated by yellow arrows. d) Respective morbidity rates of DR among diabetic C57BL/6J mice at the fourth and eighth weeks following different treatments. e) OCT images showing the structure and morphology of the retina. The red line indicates a scanning section of the murine retina, the red rectangle shows a partial view of the retina, and the yellow arrow indicates obvious edema. f) Quantitative analysis of the difference in retinal thickness at the site of edema (*n* = 5). ^*^ *p* < 0.05, ^***^ *p* < 0.001, test group versus Model group; ^###^ *p* < 0.001, Model group versus Con group. The data are presented as the means ± SDs, and statistical analysis was performed using one‐way ANOVA.

To evaluate the therapeutic effect of the CSGC hydrogel in vivo, we established diabetic animal models using intraperitoneal injection of streptozocin (STZ) and a high‐fat diet (HFD) in C57BL/6 mice (**Figure** **5**a). Before the end of the experiment, the blood glucose of all diabetic mice increased sharply, and no significant differences in average blood glucose levels were noted among the Model, Cu‐PEI, Cu‐PEI/si, and CSGC groups (Figure 5b; Figure S5a, Supporting Information). Fundus imaging at initial detection revealed the presence of small glass warts in the optic disc of the Model group, indicating the successful establishment of the murine DR model (Figure 5c). Over time, these warts in the Model group merged, enlarged, and even became blurred at the fourth, sixth, and eighth weeks, indicating the presence of severe exudate. Similar phenomena were observed in the groups treated with naked siMyD88 and Cu‐PEI USNPs. In the Cu‐PEI/siMyD88 group, small glass warts appeared in the 4^th^ week but seemed to disappear by the sixth week in response to the administration of Cu‐PEI/siMyD88 NPs at the end of the fourth week. In contrast, the CSGC group exhibited only a small number of warts throughout the eight weeks, suggesting that the glucose‐responsive hydrogel possesses long‐term treatment properties. A 28.6% increase in the morbidity rate in the CSGC group was noted compared to a 42.9% increase in the Model group from the fourth to the eighth week (Figure 5d). Furthermore, optical coherence tomography (OCT) was performed prior to sacrificing the mice. OCT, an ophthalmic diagnostic technique, offers noninvasive, high‐resolution imaging at the cellular level and rapid imaging speed and addresses the challenge of in vivo retinal and corneal studies.^[^ ^36^ ^]^ OCT revealed significant retinal edema in the model and naked siMyD88 groups, whereas no notable differences in retinal degeneration were observed in the other groups (Figure 5e). Quantitative analysis revealed variations between the thickest and thinnest parts of the optic disc, indicating that the Model group exhibited the most pronounced local retinal swelling (Figure 5f). Compared to the Model group, the CSGC hydrogel group exhibited significantly less swelling, approaching the levels observed in the normal group. The thicknesses of the RPE layer and choroid were also evaluated (Figure S5b, Supporting Information). Consistent with the pathological observations, the RPE layer and choroid were severely impaired in the model and naked siMyD88 groups, whereas the CSGC hydrogel effectively reversed this phenomenon.

**Figure 6:**
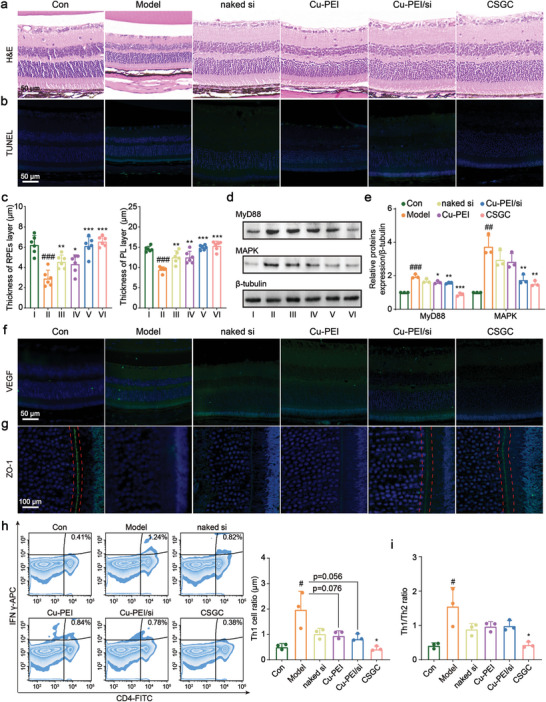
The CSGC hydrogel remodeled the RME in diabetic C57BL/6 mice. a) H&E and b) TUNEL staining; scale bar: 50 µm. c) Quantitative analysis of the thickness of the RPE layer and GCL in the retina (*n* = 6). ^*^ *p* < 0.05, ^**^ *p* < 0.01, ^***^ *p* < 0.001, test group versus Model group; ^###^ *p* < 0.001, Model group versus Con group. d) Western blotting and e) quantitative analysis of MyD88 and MAPK protein expression levels in eye tissues after exposure to different treatments. I: Con, II: Model, III: naked siMyD88, IV: Cu‐PEI USNPs, V: Cu‐PEI/siMyD88 NPs, and VI: CSGC hydrogel (*n* = 3). ^*^ *p* < 0.05, ^**^ *p* < 0.01, ^***^ *p* < 0.001, test group versus Model group; ^##^ *p* < 0.01, ^###^ *p* < 0.001, Model group versus Con group. f) VEGF (blue: DAPI; green: VEGF^+^) and g) ZO‐1 (blue: DAPI; green: ZO‐1^+^) expression in the retinas of C57BL/6 mice. The red rectangle shows the obvious dense tight junction bands formed by ZO‐1. The scale bars are 50 and 100 µm. h) Flow cytometry showing the Th1 cell ratio in eye tissues extracted from mice receiving the indicated treatments (*n* = 3). ^*^ *p* < 0.05, test group versus Model group; ^#^ *p* < 0.05, Model group versus Con group. i) The ratio of Th1 cells/Th2 cells in the different groups determined using flow cytometry (*n* = 3). ^*^ *p* < 0.05, test group versus Model group; ^#^ *p* < 0.05, Model group versus Con group. All the data are presented as the means ± SDs, and statistical analysis was performed using one‐way ANOVA.

In addition, cryosectioning and hematoxylin and eosin (H&E) staining of eye tissues revealed that the retina in the Model group was thinnest, indicating severe impairment of the physiological structure and morphology of the retina (**Figure** **6**a). Additionally, a TUNEL staining assay demonstrated that the CSGC hydrogel protected retinal cells from apoptosis (Figure 6b). Visualization of distinct cellular layers within the retina and related quantitative analysis (Figure 6c; Figure S5c,d, Supporting Information) revealed that the CSGC hydrogel exhibited the most effective therapeutic efficacy in maintaining the thickness of the RPE layer, thus preserving its compact structure and repairing the impaired retinal barrier. Similar conclusions were also obtained from the thickness measurements of the photoreceptor layer (PL), outer nuclear layer (ONL), inner nuclear layer (INL), and inner plexiform layer (IPL). Moreover, retinal ganglion cells, which are crucial for transmitting signals to the optic nerve, suffered significant injury in DR, as evidenced by an ≈50% reduction in the thickness of the ganglion cell layer (GCL) in the Model group (Figure 6c). The loss and degeneration of retinal ganglion cells directly leads to an irreversible decrease in vision.^[^ ^37^ ^]^ Both the CSGC hydrogel and Cu‐PEI/siMyD88 NPs exhibit a protective effect on ganglion cells, with the CSGC hydrogel showing superior efficacy, suggesting that sustained ocular drug delivery systems are more effective in protecting the retina and vision.^[^ ^38^ ^]^

### 2.12 Remodeling the RME Using the CSGC Hydrogel in Diabetic Mice

Although the therapeutic effects of CSGC hydrogels have been previously investigated in a murine model of DR, the underlying mechanism remains to be confirmed. MyD88 has been widely recognized for its central role in immune regulation in various diseases. However, we hypothesize that the therapeutic impact of *Myd88* silencing could be amplified in the presence of Cu‐PEI USNPs. Therefore, to further explore the therapeutic mechanisms of the CSGC hydrogel, we conducted mechanistic experiments to investigate its DR‐protective effect in vivo.

Initially, we examined the expression of MyD88 and downstream MAPK signaling. The results indicated that the CSGC hydrogel significantly suppressed the expression of these genes compared to that in the Model group (Figure 6d,e). Interestingly, the intensity of MyD88 expression in the Con group was greater than that in the CSGC group but lower than that in the Cu‐PEI/si group, and this effect can potentially be attributed to the sustained release behavior of the CSGC hydrogel. Subsequent immunofluorescence assays targeting VEGF and ZO‐1 were performed. The CSGC hydrogel effectively inhibited VEGF expression and dense, well‐defined tight junction bands of ZO‐1 were prominently observed in the Con, Cu‐PEI/si, and CSGC groups, supporting the notion that the CSGC hydrogel prevents secondary BRB injury (Figure 6f,g).

Furthermore, as a downstream effect of ROS‐primed NLRP3 inflammasome activation and pyroptosis, the secretion of mature IL‐18 and *α*‐MSH was also assessed. The results revealed that the CSGC hydrogel significantly inhibited IL‐18 maturation and increased *α*‐MSH secretion (Figure S5e,f, Supporting Information), indicating its protective effect against primary BRB injury. Previous experiments have demonstrated that the CSGC hydrogel regulates the activation of innate immune cells, prompting us to investigate its potential influence on adaptive immune cells. Therefore, additional flow cytometry analysis was conducted to detect Th1 and Th2 cells in eye tissues, and the results demonstrated that the CSGC hydrogel significantly reduced the Th1 cell ratio, an effect not observed with other treatments (Figure 6h). Although no obvious differences were noted in the Th2 cell ratio in the groups experiencing DR progressing (Figure S5g, Supporting Information), the Th1/Th2 ratio was reduced by the Cu‐PEI/siMyD88 NPs and CSGC hydrogels (Figure 6i). Hence, it can be concluded that the CSGC hydrogel inhibits CD4^+^ T‐cell differentiation into inflammatory Th1 cells. Taken together, the results of pharmacological and mechanistic studies illustrated that the CSGC hydrogel inhibited primary and secondary BRB injury through changes in the levels of cellular and soluble molecules and in the extracellular matrix to remodel the RME and treat DR efficiently.

## 3 Conclusion

We developed a sustained glucose‐responsive CSGC hydrogel that effectively delivers siMyD88 and the potent antioxidant Cu‐PEI nanoenzyme into RPEs. Upon release, Cu‐PEI/siMyD88 NPs exhibited significant efficacy in ameliorating disturbances in the RME, involving limiting microglial cell activation, regulating CD4^+^ T‐cell differentiation, inhibiting pro‐inflammatory factors, and restoring TJ expression. The proposed innovative strategy of remodeling the RME through concurrently inhibiting primary and secondary BRB injury could be used to treat DR. Notably, although antidiabetic agents were not used in this study, we anticipate enhancing anti‐DR therapeutic outcomes by combining glucose‐responsive CSGC hydrogels with such agents. The promising therapeutic effects demonstrated in this research present an encouraging strategy for leveraging RME remodeling in the treatment of DR.

## 4 Experimental Section

### Materials and Reagents

PEI (1.2 kDa) was purchased from Sigma–Aldrich (St. Louis, MO, USA). CuCl_2_ was purchased from Meryer Chemical Technology Co., Ltd. (Shanghai, China). A LysoTracker Red kit and Hoechst 33342 were purchased from Beyotime Biotechnology (Jiangsu, China). *α*‐d‐glucopyranose, HEMA, *β*‐glucosidase, linezolid, and VX765 were purchased from Meryer (Shanghai) Biochemical Technology Co., Ltd. Con A, AIBA, and EDC were purchased from Yuanye Bio‐Technology Co., Ltd. 1,4‐dioxane was purchased from Anhui Zesheng Technology Co., Ltd.

The primers used for quantitative PCR, siMyD88, and FITC‐labeled siMyD88 were purchased from Sangon Biotech Co., Ltd (Shanghai, China). A RiboGreen assay kit was purchased from Beijing Solarbio Science & Technology Co., Ltd. The All‐in‐One First‐Strand cDNA Synthesis Kit and PerfectStart Green qPCR Kit were obtained from Transgene Biotech, Inc. (Beijing, China). A monoclonal anti‐MyD88 antibody was purchased from Boster Biological Technology Co., Ltd. (Wuhan, China). Mouse anti‐*β*‐actin and anti‐*β*‐tubulin (55 kDa) were purchased from ZSGB‐BIO Co., Ltd. (Beijing, China). Monoclonal anti‐ZO‐1 and anti‐NLRP3 antibodies were purchased from Proteintech Group, Inc. All other agents and antibodies used in this study were of the highest commercial grade available.

### Synthesis and Characterization of Cu‐PEI USNPs and Cu‐PEI/siMyD88

The synthesis of Cu‐PEI USNPs involved the following procedures. Initially, 10 mm CuCl_2_ powder was dissolved in 50 mL of deionized water and stirred for 10 min at room temperature using magnetic stirring. Subsequently, the pH of the solution was adjusted to ≈9.0 by adding a NaOH solution after the addition of a PEI aqueous solution (36 mg mL^−1^, 1.2 kDa, 25 mL) to the CuCl_2_ solution. The resulting mixture was then maintained at 80 °C in an oil bath for 12 h with constant stirring. Larger aggregates were removed by centrifugation (10000 × g, 10 min). The supernatant from the resulting samples was dialyzed using a cutoff membrane (3.5 kDa) and then lyophilized to concentrate and purify the Cu‐PEI USNPs.

### Synthesis of Cu‐PEI/siMyD88 USNPs and Agarose Gel Electrophoresis Retardation Assay

Briefly, the synthesis of Cu‐PEI/siMyD88 USNPs involved the addition of siMyD88 to the Cu‐PEI solution employing varying weight ratios of Cu‐PEI to siMyD88. The mixture was then stirred at room temperature for 30 min. The resulting Cu‐PEI/siMyD88 USNPs, characterized by different weight ratios of Cu‐PEI to siRNA (1, 0.5, and 0.33), as well as a set of negative control groups (involving naked siRNA), were loaded onto a 0.8% agarose gel. Electrophoresis was performed in 1× TAE running buffer at a voltage of 120 V for 15 min. The results were subsequently visualized and analyzed under UV light at a wavelength of 254 nm using an Imagemaster VDS thermal imaging system (Bio‐Rad, Hercules, CA, USA).

### Preparation and Characterization of GEMA

GEMA was synthesized using a typical method with slight modifications. Specifically, 700 mg of *α*‐d‐glucopyranose was dissolved in 1 mL of deionized water, followed by mixing with 6 mL of HEMA and 1 mL of 1,4‐dioxane. The mixture was incubated at 50 °C for 12 h after the addition of 360 U of *β*‐glucosidase. The progress of the reaction was periodically monitored using TLC. The reaction product was subsequently purified by column chromatography (methylene chloride:methanol = 10:1), followed by rotary evaporation at 40 °C. The products were characterized using ^1^H‐NMR (Bruker Advance 400 MHz) and an infrared spectrophotometer (TENSOR 27, Bruker, Germany).

### Preparation of the CSGC Hydrogel

Four types of CSGC hydrogels with varying initial Con A concentrations were synthesized for this study. Briefly, AAc containing carboxyl groups was covalently coupled with Con A using EDC as a classical catalyst as follows. First, 0.3 mmol of EDC and 0.15 mmol of AAc were added to 1 mL of an aqueous solution containing different amounts of Con A and stirred for 1 h at room temperature. Then, a GEMA solution was added to the resulting solution until the final concentration of GEMA reached 20 wt%. After the addition of AIBA, the mixture was irradiated at 280 nm for 1 h. Once the solution was gelled, the hydrogels were immersed in a tris(hydroxymethyl)aminomethane (Tris)‐HCl buffer solution (pH 7.5) enriched with 1 mm MnCl_2_ and 1 mm CaCl_2_, aiming to eliminate any unreacted monomers. This purification process was performed at 4 °C over a period of 12 h.

### Measurement of the Amount of Con A Leaking from CSGC Hydrogel and Swelling Ratio Detection

After removing residual chemicals and unreacted monomers, the quantification of non‐immobilized Con A was initiated. The Con A concentration that leached into the Tris‐HCl buffer was determined by measuring its absorbance at 280 nm using UV spectroscopy, taking advantage of the strong absorption properties of proteins in this range. Subsequently, the swelling ratio of the CSGC hydrogel was also evaluated. Briefly, different types of CSGC hydrogels were transferred and immersed in buffer solutions with varying glucose concentrations (0, 3, and 6 mg mL^−1^) at 4 °C. At specific time intervals, the hydrogels were carefully removed from the buffer solution. Excess surface water was gently removed using absorbent paper, and the hydrogels were weighed repeatedly. Once the mass of the hydrogels reached a constant value, the swelling ratios were calculated based on the weight of the swollen hydrogel (*W*) and the weight of the dried gel (*W*_0_), as shown in the following equation:

(1) $$Swelling\;ratio\;=\frac{W-W_{0}}{W_{0}}$$

### Glucose‐Responsive Behavior and siRNA Release Assay

As previously mentioned, a RiboGreen assay kit was used to detect free siRNA in glucose buffer. Briefly, prior to the release experiment, four types of CSGC hydrogels with mass ratios of 1:5, 1:10, 1:15, and 1:20 of siRNA to Con A‐GEMA were prepared. After gelation, the hydrogels were immersed in a Tris‐HCl buffer solution containing 1 mm MnCl_2_ and 1 mm CaCl_2_ at 4 °C for 12 h. Subsequently, the hydrogels were transferred to a 50 mL buffer solution containing 3 mg mL^−1^ glucose and maintained at 37 °C. At specific time points, 0.5 mL buffer solution was withdrawn and analyzed following the manufacturer's instructions. Importantly, 0.5 mL of fresh buffer solution was promptly replenished into the original incubation system.

### Characterization of the Morphology and Composition of the Cu‐PEI USNPs and CSGC Hydrogel

The physicochemical characteristics of the aforementioned formulations were meticulously examined and characterized using various advanced techniques. Morphological information of the Cu‐PEI USNPs was obtained through transmission electron microscopy (HT‐7700, Hitachi, Japan). The absorption spectra of the CSGC hydrogel and its constituent components were acquired using a UV–vis spectrophotometer (Agilent, Santa Clara, CA, USA). Additionally, dynamic light scattering (DLS; Zeta SIZER NANO ZS90, Malvern Ltd., Malvern, UK) was employed to probe the hydrodynamic particle size and zeta potential of the Cu‐PEI USNPs. The crystal structure of the Cu‐PEI USNPs was elucidated using powder XRD (Bruker D8 advanced diffractometer system, Germany) with a 1.54 Å Cu K*α* source. XPS of the Cu‐PEI USNPs was conducted with an ESCALab220i‐XL high‐performance electron spectrometer featuring a monochromatic Al K*α* source. Furthermore, structural morphology information and elemental mapping of the CSGC hydrogel were obtained using field emission scanning electron microscopy (Apreo S LoVac, Czech Republic).

### Viscosity, Mechanical Properties, and Degradation Characterization

Viscosity characterization was performed using a rheometer (MCR302, Anton Paar GmbH, Austria), and the results were measured at room temperature with a shear rate range of 0.1–100 s^−1^. The injectability of the CSGC hydrogel (prestained with rhodamine b solution) was assessed by injection using a needle syringe at room temperature. A DMA 242 C/1/G instrument (Netzsch, Selb, Germany) was used to evaluate the elastic and loss moduli of the hydrogels. The measurements were conducted in compression mode using a frequency of 10 Hz across temperatures ranging from 20 to 120 °C with a controlled cooling rate of 3 °C min⁻¹. The degradation assay was investigated in the artificial tear solution (0.9% NaCl, 0.15% NaHCO_3_, 0.02% boric acid, 0.5% mannitol, and 0.1% sodium hyaluronate) and sodium citrate–hydrochloric acid buffer solution (pH 5.5). The mass loss was quantitatively assessed by comparing the initial dry mass (*W*) with the subsequent mass of the hydrogel after incubation in the solutions at 37 °C, over regular time intervals. After each 2‐day interval, the hydrogels were removed from the solutions and dried in an oven at 60 °C for 6 h (*W*_d_). The degradation ratio was calculated using the following equation:

(2) $$Degradation\;ratio\;=\frac{W-W_{d}}{W}$$

### CAT‐Like Activity of Cu‐PEI USNPs

The assessment of the CAT‐like activity of Cu‐PEI USNPs involved a combination of analytical techniques, including electron spin resonance (ESR) spectroscopy, oxygen generation, and H_2_O_2_ reduction. For ESR spectroscopy, a sample solution containing 200 mm H_2_O_2_ and 0.5 mm of the water‐soluble spin label 3‐carbamoyl‐2,5‐dihydro‐2,2,5,5‐tetramethyl‐1H‐pyrrol‐1‐yloxyl (CTPO) was prepared. Various concentrations of Cu‐PEI USNPs (50, 100, 200, 400, and 800 µg mL^−1^) were introduced to the solution, and ESR spectra were recorded after a 30‐min incubation. Catalase activity was also assessed by measuring oxygen generation. Specifically, a sample solution consisting of PBS and 200 mm H_2_O_2_ was mixed with Cu‐PEI USNPs to measure the concentration of oxygen produced. To evaluate the H_2_O_2_ consumption rate, a mixture of 50 mm H_2_O_2_ and Cu‐PEI USNPs (100 µg mL^−1^) was prepared at a 1:1 ratio. The supernatant was then collected at various time points, and the absorbance at 240 nm was measured. The reaction rates for different H_2_O_2_ concentrations (ranging from 0 to 30 mm) were used to establish the Michaelis‒Menten equation. In this equation, the H_2_O_2_ concentration served as the independent variable, whereas the corresponding reaction rate was the dependent variable:

(3) $$v_{0}=\frac{v_{max}\left[S\right]}{k_{m}+\left[S\right]}\;$$

### SOD‐Like Activity of Cu‐PEI USNPs

The SOD‐like activity of Cu‐PEI USNPs was investigated utilizing ESR spectroscopy. For this purpose, a solution was prepared to contain 100 mm BMPO, 0.2 mm diethylene triamine pentaacetic acid, 10 mm xanthine, and 1 U mL^−1^ xanthine oxidase. Varying final concentrations of Cu‐PEI USNPs (50, 100, 200, 400, and 800 µg mL^−1^) were added to the solution, and ESR spectra were recorded after a 30‐min incubation period. Additionally, the ·O^2−^ inhibition rate was quantified using a SOD kit (Beijing Solarbio Science & Technology Co., Ltd.).

### Cell Culture and Cell Model Establishment

RPE cells were obtained from X‐Y Biotechnology Co., Ltd (Shanghai, China) and cultured as monolayers in DMEM/F12 supplemented with 10% FBS and penicillin/streptomycin (100 µg mL^−1^) at 37 °C and 5% CO_2_. Following preincubation with various samples for 12 h, the cells were exposed to serum‐free DMEM containing TGF‐*β*1 (10 ng mL^−1^) to induce a model of tight junction injury. Moreover, to mimic a high‐glucose environment, the cells were maintained in serum‐free DMEM supplemented with 50 mm glucose, whereas the control group cells were cultured in serum‐free DMEM supplemented with 5 mm glucose.

### Cell Viability and LDH Assay

Cell viability was evaluated using a cell counting kit (CCK‐8 kit following the manufacturer's instructions. Specifically, RPE‐19 cells were seeded in DMEM supplemented with 10% FBS at a density of 1 × 10^5^ cells mL^−1^ and cultured for 24 h. When the cells reached 40–50% confluence, they were treated with DMEM supplemented with 10% FBS and varying concentrations of CSGC hydrogel or other samples for 24 h. Subsequently, 20 µL of CCK‐8 reagent was added to each well, and samples were incubated for 4 h. The optical density at 450 nm was measured using a microplate spectrophotometer to determine the relative cell viability.

To assess cell membrane integrity after incubation with Cu‐PEI and Lipo2000, an LDH assay was conducted. Briefly, RPE cells were cultured in 96‐well tissue culture plates until ≈50% confluence. Then, the plates were incubated with different concentrations of Cu‐PEI or other reagents for 24 h. The plates were subsequently centrifuged at 300 × g for 5 min, and 60 µL of cell‐free supernatant was incubated with 30 µL of LDH substrate solution for 30 min. The absorbance at 490 nm was recorded on a microplate spectrophotometer to calculate the LDH release activity according to the manufacturer's instructions.

### Transfection and Gene Silencing Efficiency Detection

FITC‐labeled siMyD88 was purchased from Sangon Biotech (Shanghai) Co., Ltd. RPEs, cultured to 70−80% confluence, were maintained in serum‐free DMEM containing preprepared Cu‐PEI/siMyD88 NPs with various weight ratios (Cu‐PEI:FITC‐labeled siMyD88 = 1:1, 1:2 and 1:3) for 6 h, followed by incubation with DMEM supplemented with 10% FBS for 4 h. Lipo2000 was used as the positive control. Similarly, RPEs at 70−80% confluence were cultured in opti‐MEM and incubated with a complex solution of FITC‐labeled siMyD88 and Lipo2000 for 6 h, followed by incubation with DMEM supplemented with 10% FBS for 4 h. The cells were then stained using a LysoTracker Assay Kit and Hoechst 33342 according to the manufacturers’ instructions. Confocal laser scanning microscopy was employed to examine endosome production and transfection levels in the cells, and quantification was performed using ImageJ software. The gene silencing efficiency was evaluated through corresponding qPCR and Western blotting experiments.

### Real‐Time qPCR

Total RNA was extracted from cell or animal tissue samples, and cDNA synthesis was performed using a kit. Subsequently, qPCR was conducted. The relative concentration of the samples was determined by analyzing the threshold cycle number (*Ct* value) of the fluorescence signal during the PCR. To ensure data reproducibility, a minimum of three independent biological replicates were tested. The gene‐specific primers employed for qPCR are detailed in Table S1 (Supporting Information).

### Western Blotting

Proteins were extracted from cells or animal tissues using radioimmunoprecipitation assay lysis buffer. Subsequently, the extracted proteins were separated using sodium dodecyl sulfate‒polyacrylamide gel electrophoresis and transferred to poly(vinylidene fluoride) (PVDF) membranes. Next, a specific primary antibody was used to bind the target protein, and the resulting complex was detected using a fluorescence‐labeled secondary antibody. The presence of the target protein was visualized using a fluorescence imaging system and quantified based on the relative signal intensity.

### In vitro Assessment of Antioxidant Activity

The antioxidative activity of the CSGC hydrogel was evaluated by quantifying the intracellular levels of total ROS, H_2_O_2_, and •O_2_^−^. RPEs were cultured to 70–80% confluence in confocal dishes and exposed to serum‐free DMEM containing 10 ng mL^−1^ TGF‐*β*1 and various samples (Cu‐PEI, Cu‐PEI/siMyD88, CSGC hydrogel, or Rapa) for 6 h. The control group was treated with serum‐free DMEM alone. Subsequently, the cells were stained with a DCFH‐DA) probe for 30 min and washed with PBS thrice. Finally, the cells were imaged using CLSM to observe intracellular total ROS and analyzed using flow cytometry to quantify total ROS levels. A similar protocol was followed to assess the intracellular H_2_O_2_‐, and •O_2_^−^ scavenging abilities of the CSGC hydrogel via H_2_O_2_ probe (10 µm, 1 h) and DHE (10 µm, 30 min) staining, respectively.

### Flow Cytometry Analysis of Cell Apoptosis and Pyroptosis

RPEs were cultured in six‐well plates at a density of 1 × 10^5^ per well for 24 h. Upon reaching 40–50% confluence, the cells were exposed to serum‐free DMEM containing 50 mm glucose and various samples (Cu‐PEI, Cu‐PEI/siMyD88, CSGC hydrogel, or NSA) for 24 h. The cells in the low glucose group were treated with serum‐free DMEM containing 5 mm glucose alone. Subsequently, the cells were collected, stained with an Annexin V‐FITC apoptosis detection kit (Solarbio, China), and analyzed using a flow cytometer to assess cell apoptosis. A similar protocol and FLICA probe staining from a pyroptosis/caspase‐1 assay kit (Cat. #9145, ImmunoChemistry Technologies, USA) were used to measure pyroptosis.

### Transwell Co‐Culture of RPEs and Microglia

RPEs were seeded at a density of 1 × 10^5^ cells per well in transwell chambers and allowed to adhere for 24 h. Similarly, BV‐2 cells were cultured in 1640 medium supplemented with 20% FBS and 5 mm glucose in separate dishes. After a 12 h incubation of RPEs with serum‐free DMEM containing 50 mm glucose and various samples (Cu‐PEI, Cu‐PEI/siMyD88, CSGC hydrogel, or NSA), the transwell chambers were washed with PBS to remove the samples. Then, the transwell chambers containing RPEs were inserted into a dish containing BV‐2 cells. The systems were cultured in blank DMEM supplemented with 5 mm glucose for an additional 24 h. Subsequently, BV‐2 cells were harvested, stained with MHCII–FITC (Abcam #ab239229) and TMEM119‐phycoerythrin (PE) (Abcam #ab2209064), and analyzed using a flow cytometer to assess the antigen‐presenting ability of microglia.

### Immunofluorescence Assay

RPEs were seeded at a density of 1 × 10^5^ cells per well in six‐well plates. After reaching 70–80% confluence, the cells were exposed to serum‐free DMEM containing 50 mm glucose, 10 ng mL^−1^ TGF‐*β*1, and various samples for 24 h. After washing with PBS, each well was treated with 800 µL of 4% histophytic cell fixative at room temperature for 30 min. Subsequently, the cells were permeabilized using 0.5% Triton X‐100 for 10 min and blocked with 1% BSA solution for 1 h. After the blocking step, the cells were incubated with a dilution of ZO‐1 polyclonal antibody (1:2000, Proteintech) and Occludin monoclonal antibody (1:1600, Proteintech) for 24 h. After incubation with secondary antibody dilution and 4,6‐diamidino‐phenylindole (DAPI) staining, the cells were imaged using a fluorescence microscope to visualize the content of the TJs.

### Animal Experiments

All animal experiments were approved by the Medical Ethics Committee of Peking Union Medical College and performed in accordance with the ARRIVE guidelines and the National Institutes of Health regulations for the care and use of animals in research. The animal experiments were also approved by the Animal Ethics Committee of Nankai University (approval no. 2024‐SYDWLL‐000440) and abided by the laboratory animal treatment guidelines of Tianjin University (license number: SYXK (Jin) 2019‐0002). Male C57BL/6J mice (6–8 weeks old, 18–21 g) were purchased from Vital River Laboratory Animal Technology Co., Ltd. (Beijing, China) and housed in a controlled environment with regulated temperature and humidity for one week of acclimation. Following acclimation, the mice were randomly assigned to six groups, each consisting of ten mice, and were provided with either a standard chow diet or a 60% kcal D12492 high‐fat diet (HFD). In the model group, Cu‐PEI group, naked siMyD88 group, Cu‐PEI/siMyD88 group, and CSGC group, the mice received intraperitoneal injections of a 1% STZ solution (60 mg kg^−1^) once daily for five consecutive days after an 8 h nightly fast. The control group received an equivalent volume of sodium citrate solvent. The mice continued on their respective diets for an additional 10 days, and fasting blood glucose levels were measured on the 15th day. Mice with blood glucose values less than 12 were excluded from the study. Diabetic mice received intravitreal injections of various samples once every four weeks and underwent fundus imaging every two weeks. The injection dose for each group was determined to contain 0.1 mg kg^−1^ siMyD88 as a reference. Two days before concluding the experiment, retinal OCT assessments were performed on the mice after anesthesia was induced by abdominal cavity injection of ethobrom, and pupil dilation was achieved using sodium hyaluronate eye drops. At the end of the study, the animals were fasted overnight for blood glucose measurement and then were euthanized for eyeball collection. The collected tissues were subjected to various analyses, including flow cytometry, immunohistochemistry, immunofluorescence, Western blotting, and other relevant assays.

### Statistical Analysis

The data are presented as the mean ± standard deviation (SD). Statistical analysis of the data was performed using one‐way analysis of variance (ANOVA) followed by the Bonferroni post‐hoc correction to determine the significant differences among the groups. Statistical significance was calculated using SPSS 17.0 (SPSS, IBM Corp., Armonk, NY, USA). *p* values are denoted as (^*^) for *p* < 0.05, (^**^) for *p* < 0.01, and (^***^) for *p* < 0.001, with all of these values considered statistically significant unless otherwise stated. The corresponding statistical details of the experiments are also provided in the respective figure legends. In these legends, “n” denotes the number of samples within the animal experiment groups or various molecular biological analyses.

## Conflict of Interest

The authors declare no conflict of interest.

## Author Contributions

Y.Z. and C.Z. contributed equally to this work. N.L. and C.Z. designed the experiments. Y.Z. synthesized the materials, completed the in vitro and in vivo experiments, and wrote the original draft. Z.S. analyzed the data and polished the draft. Z.H. polished the draft. H.J., Z.S., and Y.D. assisted with the in vivo experiments. S.W. and Z.Q. assisted with the in vitro experiments. All the authors approved the final version of the manuscript.

## Supporting information

Supporting Information

## Data Availability

The data that support the findings of this study are available from the corresponding author upon reasonable request.
