# Supplementary material for: A Glucose‐Responsive Hydrogel Inhibits Primary and Secondary BRB Injury for Retinal Microenvironment Remodeling in Diabetic Retinopathy
Source: Adv Sci (Weinh). 2024 Jun 21;11(32):2402368. doi: 10.1002/advs.202402368 (PMC11348052; doi:10.1002/advs.202402368)
Supplement: Supplementary file 1 — Supporting Information [file ADVS-11-2402368-s001.docx]

Supporting Information

A Glucose-Responsive Hydrogel Inhibits Primary and Secondary BRB Injury for Retinal Microenvironment Remodeling in Diabetic Retinopathy

*Yue Zhou^a,d†^, Chan Zhao^b,c†^, Zhiyuan Shi^a^, Zbynek Heger^e^, HuaQing Jing^a^, Zhengming Shi^b,c^, Yunsheng Dou^a^, Siyu Wang^a^, Zitong Qiu^a^, Nan Li^a*^*

^a^ Tianjin Key Laboratory of Drug Delivery & High-Efficiency, School of Pharmaceutical Science and Technology, Tianjin University, Tianjin, China.

^b^ Department of Ophthalmology, Peking Union Medical College Hospital, Chinese Academy of Medical Sciences, Beijing, China.

^c^ Key Laboratory of Ocular Fundus Diseases, Chinese Academy of Medical Sciences & Peking Union Medical College, Beijing, China.

^d^ Department of Pharmacy, Tianjin Union Medical Center, Nankai University, Tianjin, China.

^e^ Department of Chemistry and Biochemistry, Mendel University in Brno, Brno, Czech Republic

^*^(N. Li) Corresponding author Email address: linan1985@tju.edu.cn.

^†^These authors contributed equally to this work.

This word file includes:

Figure S1 to S5

Table S1

**
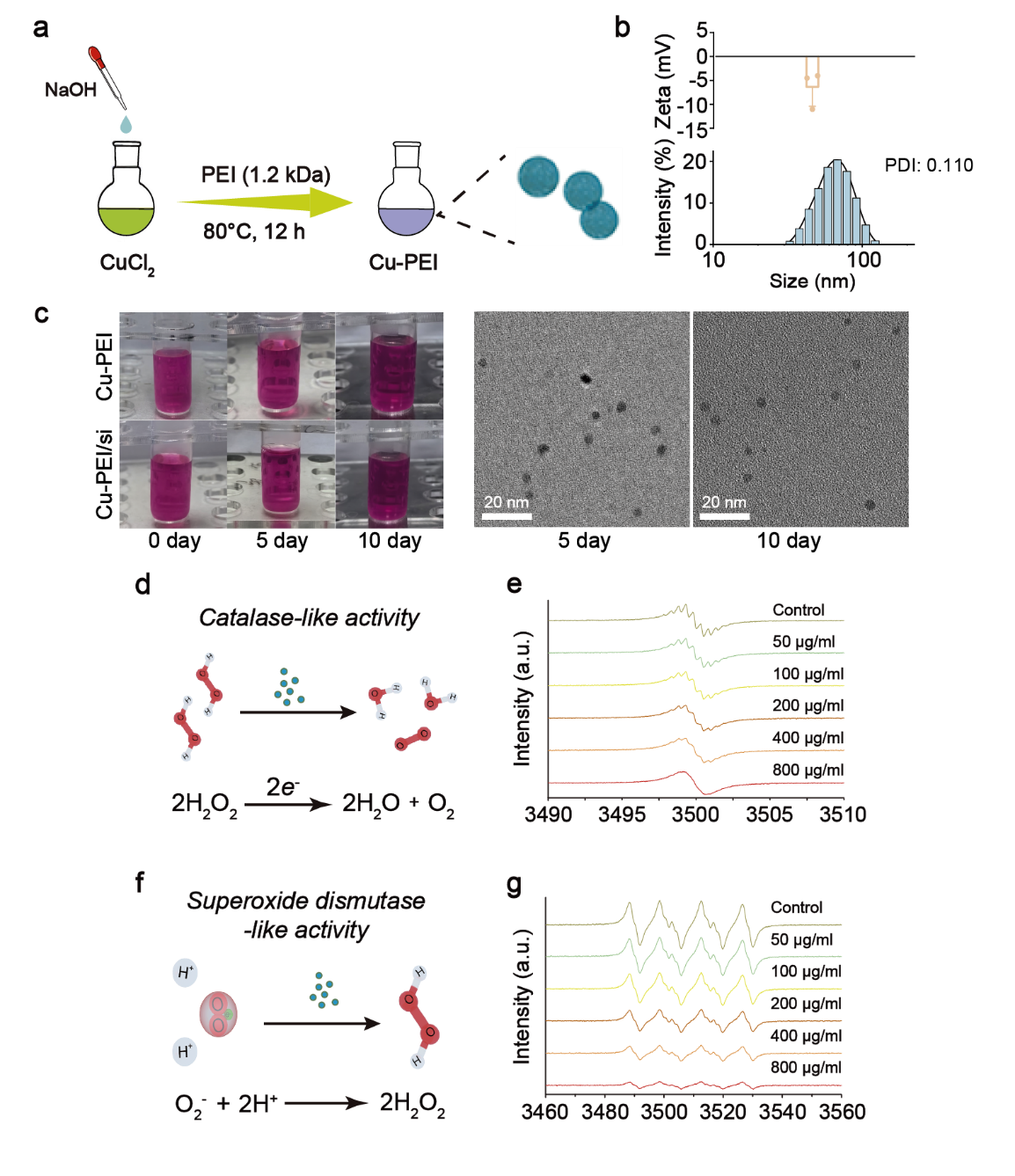
**

**Figure S1.** (**a**) Schematic illustration of the Cu-PEI USNP synthesis method. (**b**) Size and zeta potential of Cu-PEI/siMyD88 NPs measured using DLS (n=3). (**c**) Digital photos and TEM images of Cu-PEI USNPs and Cu-PEI/siMyD88 NPs in DMEM (containing 10% FBS) after 10 days; scale bar: 20 nm. (**d**) Schematic representation of the catalase-like activity of Cu-PEI USNPs. (**e**) ESR spectra demonstrating H_2_O_2_ scavenging for different concentrations of Cu-PEI USNPs. (**f**) Schematic representation of the superoxide dismutase-like activity of Cu-PEI USNPs. (**g**) ESR spectra demonstrating ·O2^-^ scavenging for different concentrations of Cu-PEI USNPs.


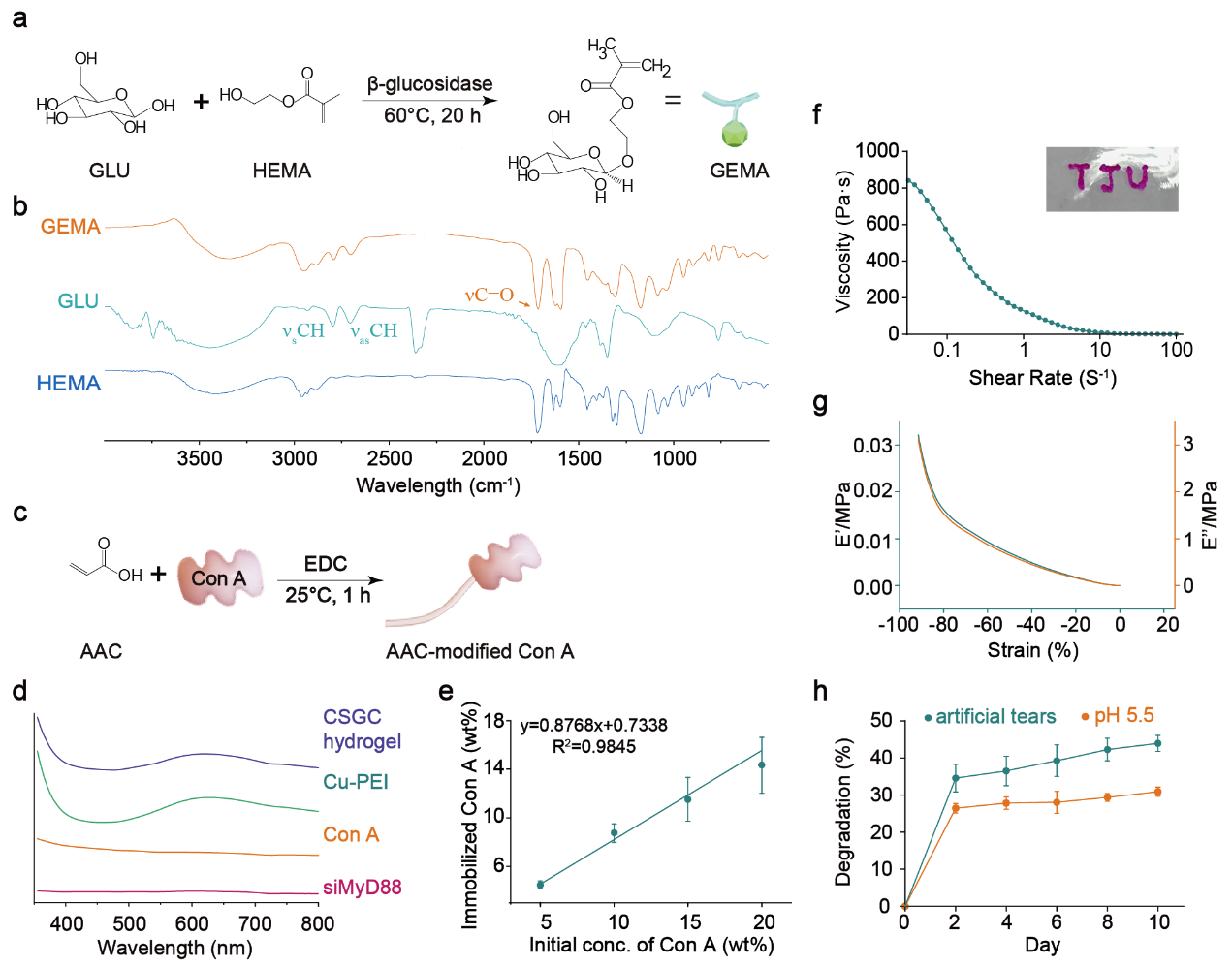


**Figure S2.** (**a**) Synthetic route for GEMA. (**b**) FT-IR spectra of GEMA, GLU and HEMA within the 4000 − 400 cm^-1^ region. (**c**) Synthetic route for AAC-modified Con A. (**d**) UV–*vis* spectra of solutions of the CSGC hydrogel, Cu-PEI USNPs, Con A and siMyD88. (**e**) Changes in the relative absorbance of H_2_O_2_ upon the addition of Cu-PEI USNPs at different times (n=6). (**f**) Viscosity of the CSGC hydrogel at shear rates ranging from 0.1 to 100 s^−1^. Inset: photographs of the injectability of the CSGC hydrogel (prestained with rhodamine b solution) through a needle. (**g**) DMA analysis of the CSGC hydrogel. (**h**) Degradation trend of the CSGC hydrogel in artificial tears and pH 5.5 buffer (n=3). The data are presented as the means ± SDs.


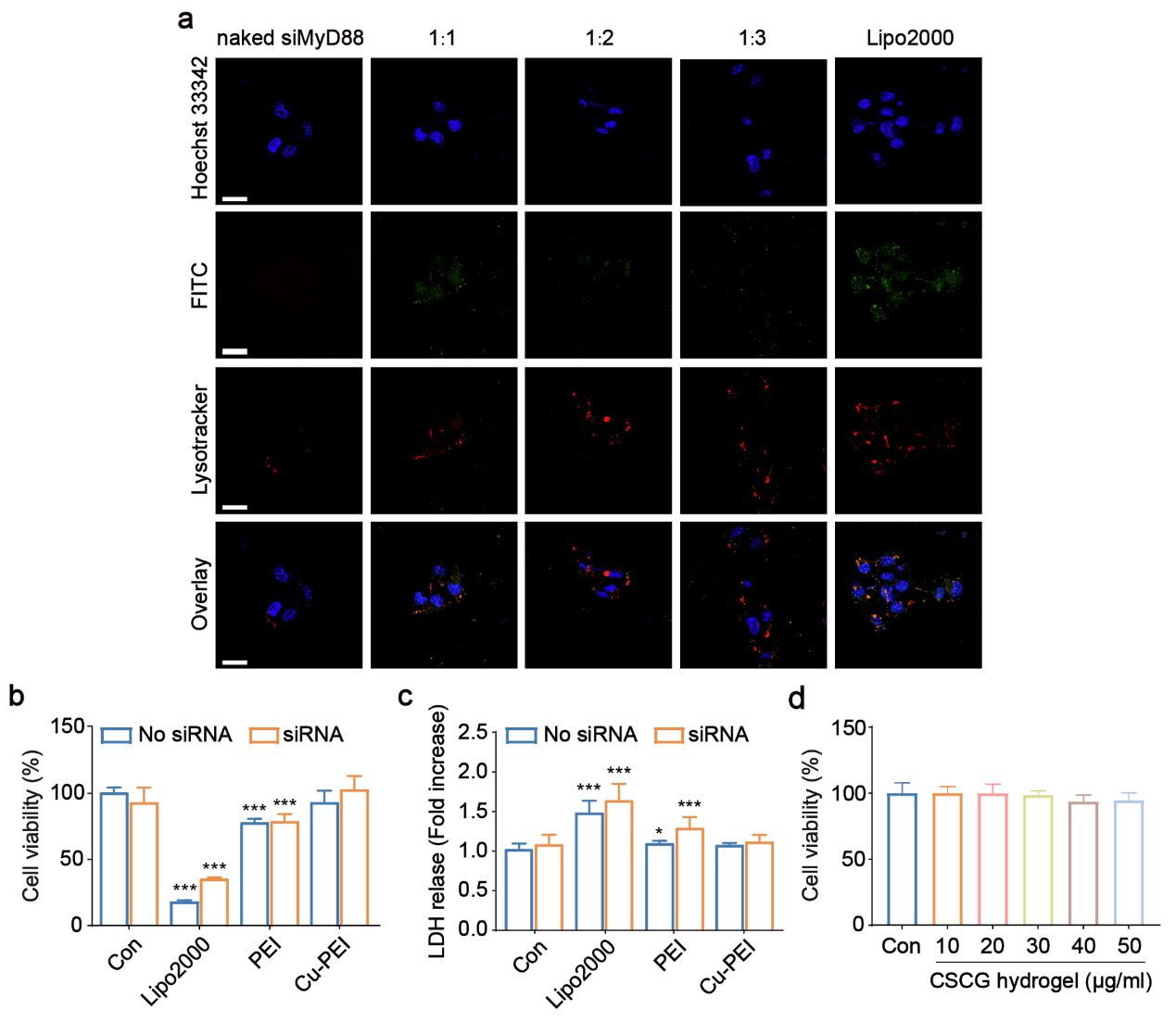


**Figure S3.** (**a**) Confocal microscopy images showing the efficiency of internalization and endosomal escape of Cu-PEI/FITC-labeled siMyD88 NPs after treatment with different weight ratios of Cu-PEI and siRNA in RPEs for 6 h (green: FITC-labeled siMyD88; red: LysoTracker; blue: DAPI); scale bars: 25 µm. (**b**) Cytotoxicity assay of RPEs after incubation with Lipo2000, PEI, and Cu-PEI NPs in the presence or absence of the same dose of siMyD88 for 24 h (n=8). ^***^p < 0.001, test group *vs.* Con group. (**c**) LDH assay of RPEs after incubation with Lipo2000, PEI, or Cu-PEI NPs in the presence or absence of the same dose of siMyD88 for 24 h (n=8). ^*^p < 0.05, ^***^p < 0.001, test group *vs.* Con group. (**d**) RPE cell viability after treatment with CSGC hydrogels at different concentrations for 24 h (n=8). All the data are presented as the means ± SDs, and statistical analysis was performed using one-way ANOVA.


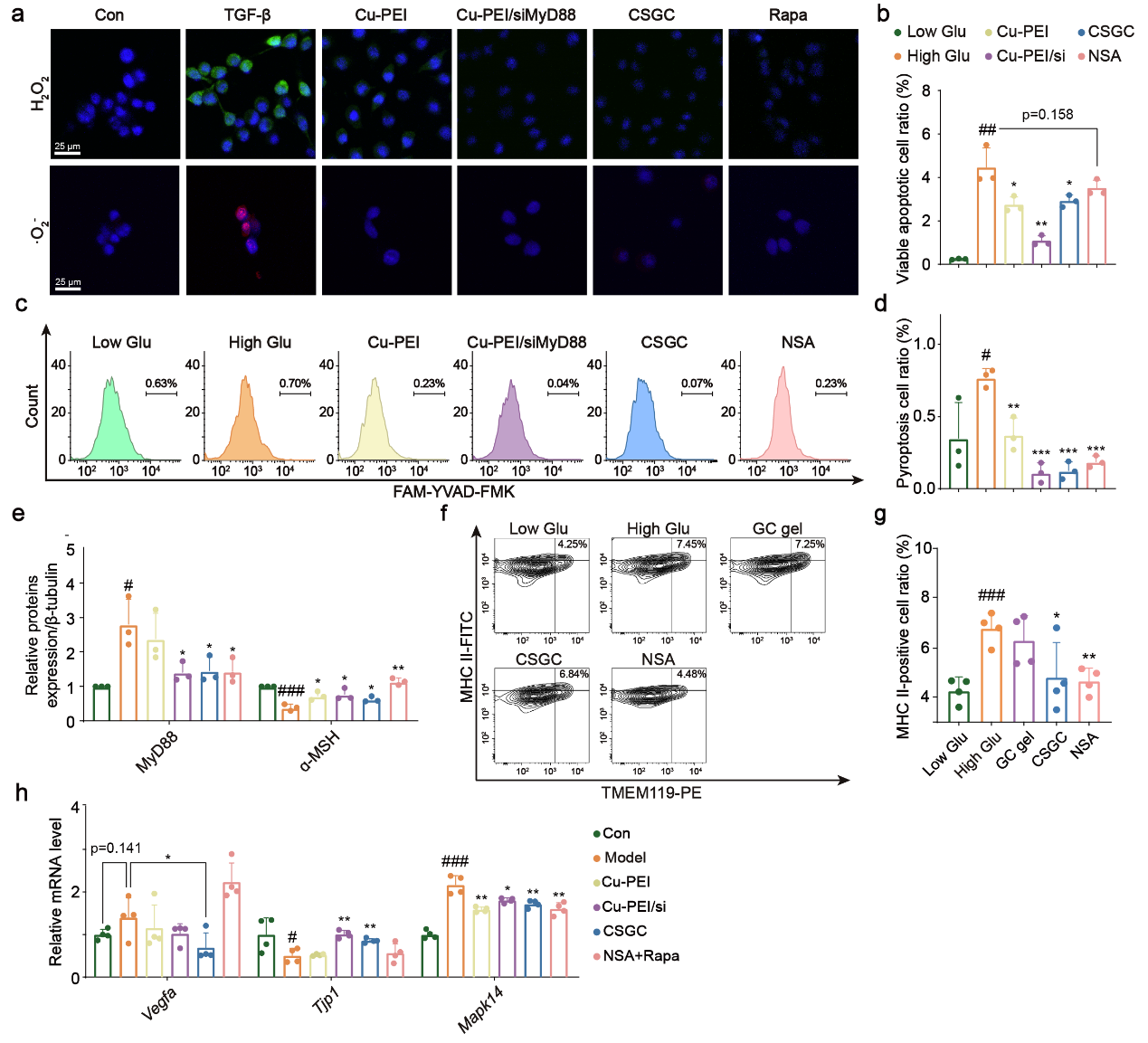


**Figure S4.** (**a**) CLSM micrographs of total intracellular ROS, H_2_O_2_ and ·O2^-^ in RPEs after different treatments; scale bar, 25 μm. (**b**) Quantitative analysis of the percentage of viable apoptotic cells (n=3). ^*^p < 0.05, ^**^p < 0.01, test group *vs.* High Glu group. ^##^p < 0.01, High Glu group *vs.* Low Glu group. (**c**) Flow cytometry assay and (**d**) quantitative analysis of pyroptotic RPEs after different treatments and related analysis (n=3). ^**^p < 0.01, ^***^p < 0.001, test group *vs.* High Glu group; ^#^p < 0.05, High Glu group *vs.* Low Glu group. (**e**) Quantitative analysis of MyD88 and α-MSH expression in RPEs after different treatments (n=3). ^*^p < 0.05, ^**^p < 0.01, test group *vs.* Model group; ^#^p < 0.05, ^###^p < 0.001, Model group *vs.* Con group. (**f**) Flow cytometry analysis of the antigen-presenting ability of microglia after different treatments and (**g**) related quantitative analysis (n=4). ^*^p < 0.05, ^**^p < 0.01, test group *vs.* High Glu group; ^###^p < 0.001, High Glu group *vs.* Low Glu group. (**h**) Quantitative analysis of *Vegfa*, *Tjp1* and *Mapk14* mRNA levels in RPEs after different treatments (n=4). ^*^p < 0.05, ^**^p < 0.01, test group *vs.* Model group; ^#^p < 0.05, ^###^p < 0.001, Model group *vs.* Con group. All the data are presented as the means ± SDs, and statistical analysis was performed using one-way ANOVA.


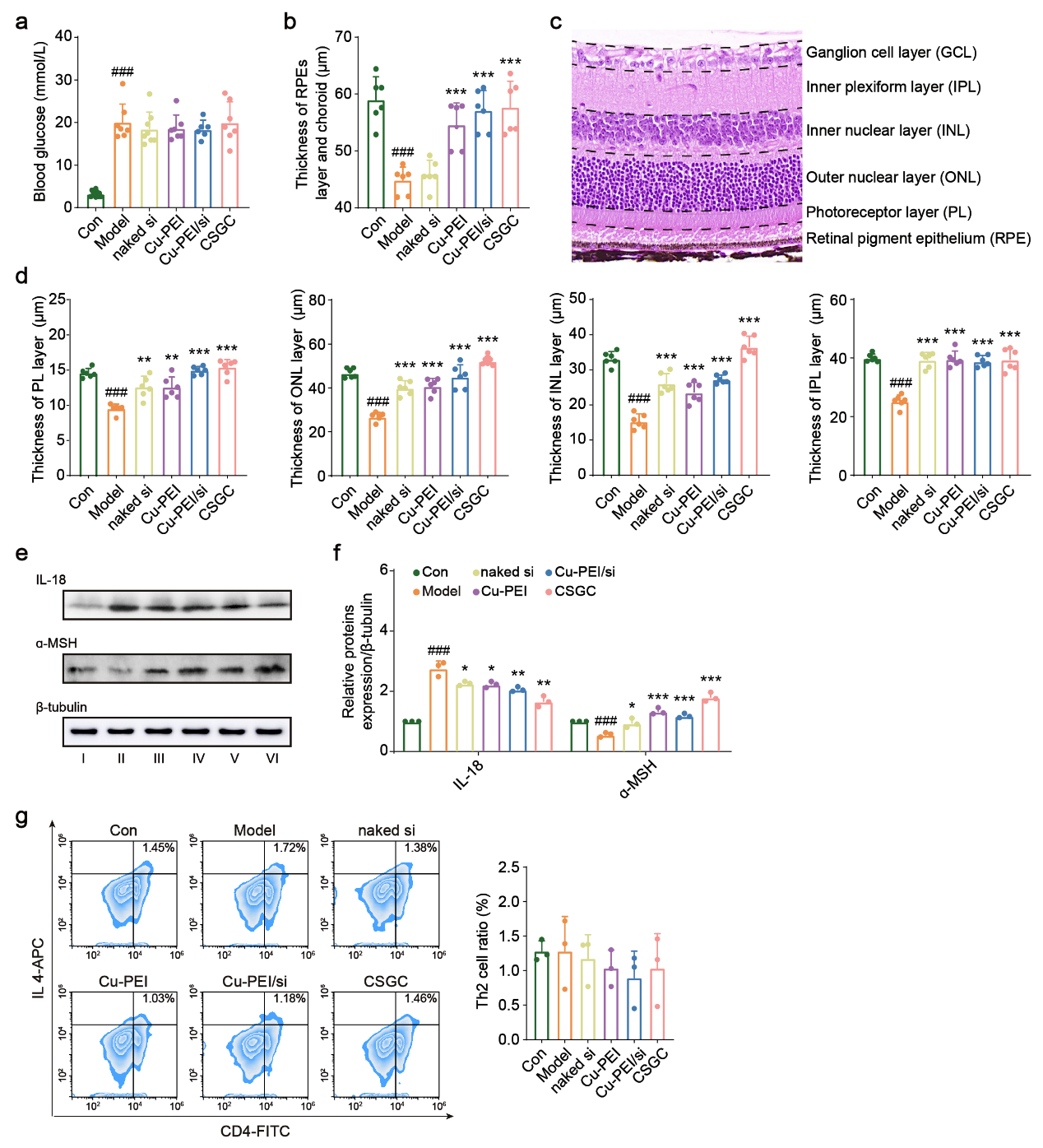


**Figure S5.** (**a**) Blood glucose levels of C57BL/6J mice at the 8^th^ week after different treatments (n=10 for the Con group; n=7 for the Model, Cu-PEI and CSGC groups; n=8 for the naked siMyD88 group; and n=6 for the Cu-PEI/si group). ^###^p < 0.001, Model group *vs.* Con group. (**b**) Quantitative analysis of the thickness of the RPE layer and choroid (n=6). ^***^p < 0.001, test group *vs.* Model group; ^###^p < 0.001, Model group *vs.* Con group. (**c**) Illustration of distinct cellular layers within the retina. (**d**) Quantitative analysis of the thickness of the PL, ONL, INL and IPL in the retina (n=6). ^***^p < 0.001, test group *vs.* Model group; ^###^p < 0.001, Model group *vs.* Con group. (**e**) Western blotting and (**f**) quantitative analysis of IL-18 and α-MSH levels in eye tissues from C57BL/6J mice after different treatments (n=3). ^*^p < 0.05, ^**^p < 0.01, ^***^p < 0.001, test group *vs.* Model group; ^##^p < 0.01, ^###^p < 0.001, Model group *vs.* Con group. (**g**) Flow cytometry showing the Th2 cell ratio in eye tissues extracted from mice receiving the indicated treatments (n=3). ^*^p < 0.05, test group *vs.* Model group; ^#^p < 0.05, Model group *vs.* Con group. All the data are presented as the means ± SDs, and statistical analysis was performed using one-way ANOVA.

Table S1. Primers for qPCR

| Primer | Sequence (5' to 3') |
| --- | --- |
| mice-*Myd88*-F | ATCGCTGTTCTTGAACCCTCG |
| mice- *Myd88*-R | CTCACGGTCTAACAAGGCCAG |
| mice-*Tjp1*-F | GCTTTAGCGAACAGAAGGAGC |
| mice-*Tjp1*-R | TTCATTTTTCCGAGACTTCACCA |
| mice-*vegfa*-F | CTGCCGTCCGATTGAGACC |
| mice- *vegfa*-R | CCCCTCCTTGTACCACTGTC |
| mice-*Mapk14*-F | TGACCCTTATGACCAGTCCTTT |
| mice- *Mapk14*-R | GTCAGGCTCTTCCACTCATCTAT |
| mice-*Actb*-F | GGCTGTATTCCCCTCCATCG |
| mice-*Actb*-R | CCAGTTGGTAACAATGCCATGT |
